# Hydration control in molecular salts through counterion selection

**DOI:** 10.1038/s41467-026-74560-5

**Published:** 2026-06-18

**Authors:** Henry A. Holleb, Natalie E. Pridmore, Amy V. Hall, Pablo Martinez-Bulit, Aurora J. Cruz-Cabeza

**Affiliations:** 1 Department of Chemistry, Durham University, Durham, UK; 2 The Cambridge Crystallographic Data Centre, Cambridge, UK

**Keywords:** Chemical physics, Crystal engineering, Cheminformatics

## Abstract

Predicting when and why molecular salts form hydrates has remained a significant problem in solid-state chemistry, despite its impact on the stability and performance of industrially relevant materials. Here, we identify counterion charge localization as a key determinant of hydrate formation in molecular salts, providing a framework for predicting and controlling solid-state hydration. By analyzing over 31,000 crystal structures and performing electron density calculations, we show that delocalized counterion charge suppresses hydrate formation, while localized charge promotes it. This principle makes hydration propensity a more tuneable property (from ~80% to <10%) through considered counterion selection. Guided by these insights and the Δp*K*_a_ rule, we experimentally demonstrate rational control of hydration in salts of 4-aminoacetanilide. Our results establish a direct link between molecular electrostatics and crystallization outcomes, affording predictive design of molecular salts with tailored hydration behavior.

## Abstract (web summary)

Predicting when and why molecular salts form hydrates has remained a significant problem in solid-state chemistry, despite its impact on the stability and performance of industrially relevant materials. Here, the authors identify counterion charge localization as a key determinant of hydrate formation in molecular salts.

## Introduction

Crystallization is a fundamental process across chemical industries, enabling the purification and isolation of compounds^1^. Despite its central role in chemical research, development and manufacturing, crystallization remains poorly understood, with its outcomes notoriously difficult to anticipate^2–5^. Among the most unpredictable phenomena is the formation of solvates, crystals that incorporate solvent molecules into their crystal structure, with hydrates being particularly common^6–14^.

There are several compelling reasons to investigate hydrate formation. First, water is a prolific solvate former due to its small size, simple geometry, and strong dipole moment^7,10–12^. Second, water is ubiquitous and can be absorbed from the atmosphere by many solvents, leading to unexpected hydrate formation even when crystallization occurs from nominally non-aqueous media^15,16^. Once incorporated into crystals, water molecules frequently become mobile in response to changes in temperature, pressure, or humidity, often resulting in solid-form changes involving full or partial dehydration^7,10,11^. Importantly, hydrates of various stoichiometries, their polymorphs, non-stoichiometric hydrates, anhydrates, and dehydrated forms all display distinct physicochemical properties, complicating product formulation^7,12,17–20^.

Many molecular-based consumer products are formulated as crystalline salts, including food additives (e.g., sodium glutamate), agrochemicals (e.g., glyphosate isopropylamine), pharmaceuticals (e.g., fluoxetine hydrochloride), and organic conductors and semiconductors (e.g., the charge-transfer salt tetrathiafulvalene–tetracyanoquinodimethane). Notably, around 50% of drugs are salts, as this often improves solubility and related properties^21–27^; and charge-transfer salts constitute a central class of materials in organic conductors and semiconductors. Salt design and formulation are therefore critical across multiple industries. Given an active molecular ingredient (AMI) of interest, salt design involves two fundamental steps: (i) selection of a counterion (chemical design) and (ii) crystallization of the salt (materials design).

**Fig. 1:**
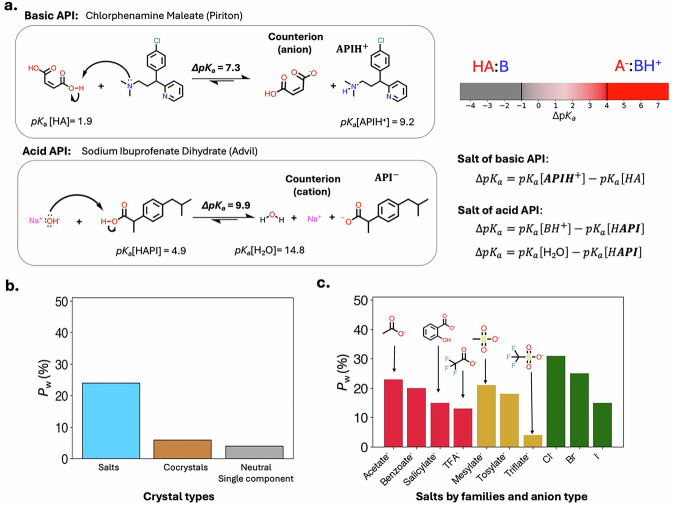
Salt formation and hydration trends. **a** Examples of salt formation for basic and acidic APIs (exemplified with Piriton and Advil salts) and Δp*K*_a_ rule (right). **b** Hydration probability (*P*_*w*_ in %) of molecular crystals by type. **c** Hydration probability (*P*_*w*_ in %) of crystalline salts classified by counterion type (TFA: trifluoroacetate).

Typically, the AMI is first produced as a neutral compound that is subsequently ionized to form the salt. For charge-transfer salts, ionization is achieved by pairing the AMI with a counter-component capable of donating or accepting an electron to/from the AMI, so that mixing results in partial charge transfer and salt formation. Here, the relative ionization potential of one component and the electron affinity of the other drive ionization. For salts involving acidic or basic AMIs (as is the case for most active pharmaceutical ingredients, APIs), salt formation is achieved by pairing the AMI with a counter-compound capable of proton transfer. Figure 1a illustrates salt design for basic and acidic APIs paired with suitable acid and base counter-components^22^. The likelihood of salt formation between an acid and a base pair can be estimated using the empirical Δp*K*_a_ rule, which compares the p*K*_a_ of the protonated base with that of the acid (Fig. 1a, right). When Δp*K*_a_ > 4, salt formation is favored; when −1 <Δp*K*_a_ < 4, the outcome depends on the crystal environment; and when ΔpKa <−1, salts are unlikely to form^28^.

Designing salts involves more than simply selecting a counterion based on ionization/electron accepting capabilities or applying the Δp*K*_a_ rule. The choice of counter-compounds and the resulting ionized counterions profoundly influences the salt’s crystallization behavior, crystal structure, physicochemical properties (e.g., melting point, hygroscopicity, mechanical strength), and manufacturability^29–32^. Factors, such as counterion size, shape, hydrogen-bonding ability, and charge delocalization, affect a salt’s properties in ways that are often unpredictable^29,30,33,34^. In practice, beyond ionization and p*K*_a_ considerations, salt ‘design’ is largely driven by brute-force screening, testing numerous pairings and crystallization conditions to generate as many salts as possible, followed by characterization and property evaluation^29–32^. Thus, salt design remains a multidimensional optimization problem, still dominated by experimental trial-and-error approaches.

The Cambridge Structural Database (CSD), containing over 1.3 million small-molecule crystal structures, consolidates global crystallographic knowledge in one resource^35^. Numerous studies have used the CSD to investigate hydrate formation in molecular crystals. Infantes *et al*. investigated water–water interactions in crystals describing commonly observed assemblies (rings, chains, and sheet motifs), while Gillon *et al*. quantified the most frequent water environments in crystals -with three hydrogen bonds being the most common^36,37^. Later, Infantes *et al*. reported that polarity and hydrogen-bond donors and acceptors increase a compound’s potential to form hydrates^38^. Recent studies making use of the CSD Python Application Programming Interface (CSD Python API) have enabled more complex analyses of hydrates in the CSD, with Werner and Swift reporting structural relationships between hydrate–anhydrate pairs^39,40^. Regarding salts, studies by Aakeroy *et al*. and Cruz-Cabeza *et al*. have shown that crystals containing ionic species hydrate significantly more frequently than those composed of uncharged molecules^41,42^. Indeed, around 21% of salts form hydrates compared to just 6% of cocrystals and 4% of neutral single-component crystals (Fig. 1b). The mechanistic reasons behind the heightened hydration propensity of crystals containing ionic species remain unclear, revealing a crucial gap in our understanding.

To address this gap, we performed a systematic analysis of salts in the CSD, classifying them by common counterions. While salts overall are significantly more prone to hydration than neutral crystals (Fig. 1b), we find that hydration propensity varies markedly depending on the nature of the counterion (Fig. 1c). In particular, we observe that charge localization on the counterion plays a critical role: counterions capable of delocalizing charge form significantly fewer hydrates than those with localized charge (Fig. 1c). Motivated by this observation, we investigated the structural features governing charge localization in counterions and their relationship to hydrate formation. Using an extensive structural dataset and electron density calculations, we uncover a principle that enables the prediction of hydration probabilities for molecular salts. We demonstrate this principle experimentally by designing salts of a compound with previously unknown salt formation and hydration behavior. This work establishes design rules linking counterion selection to hydration behavior in molecular salts.

## Results and discussion

**Fig. 2:**
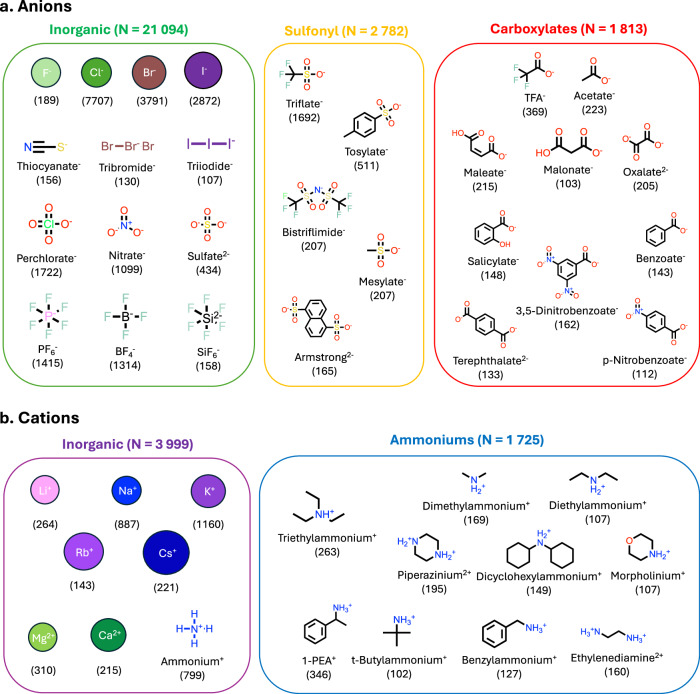
Families of common counterion types in the CSD. 31,401 salts were classified into five main families based on the structure of their most common counterion. The number of crystal structures in each family is given in parentheses. **a** The anionic families include sulfonyl anions, carboxylate anions, and inorganic anions. **b** The cationic families contain inorganic cations, and ammonium cations (1-PEA: 1-phenylethylammonium).

Salts in the CSD were surveyed, and common counterions were identified and classified into families. All counterions appearing in at least 100 crystal structures were extracted by clustering based on SMILES representations, resulting in 71 common counterions split into four types (Fig. S1). Of these, 22 were excluded from further analysis because they were either (i) too bulky to allow direct interaction between water and the charged site (6 ions, e.g., tetrabutylammonium), or (ii) contained additional (non-intramolecular) hydrogen-bond donors beyond the charged XH^+^ group (16 ions, e.g., citrate), leading to more complex and less predictable hydration behavior. The remaining 49 counterions were considered for analysis, of which 46 were classified into five distinct families, with three left unclassified (pyridinium, picrate, and DMAP). This yielded a dataset of 46 common counterions across more than 31,000 molecular salts, grouped as inorganic anions (13 counterions, with thiocyanate considered as an inorganic due to its similarity to iodide), sulfonyl anions (5), carboxylate anions (10), inorganic cations (8), and ammonium cations (10), with all counterions analyzed shown in Fig. 2.

The most prevalent salt families in the CSD (classified by counterion type; Fig. 2) are those containing inorganic anions (21.1k structures) or inorganic cations (4.0k), followed by sulfonyl anions (2.8k), carboxylate anions (1.8k), and ammonium cations (1.7k). The most common anionic counterions are chloride (7707 structures), bromide (3791), iodide (2872), and triflate (1692), while the most common cationic counterions are potassium (1160), sodium (887), ammonium (799), and magnesium (310). While many of these counterions are widely used in pharmaceutical salt formulation, others serve various roles in different fields. For example, 1-phenylethylammonium (1-PEA^+^) is commonly used in studies of chiral systems, while succinate salts are widely used as food additives^43–46^.

Next, we calculated the experimental propensity of hydration (*P*_*w*_, %) for salts by counterion type using CSD data. This probability was determined by classifying each salt structure as anhydrous or hydrated (if it contained water) and calculating the fraction of hydrated structures (*N*_Hydrates_) over the total number of salts (*N*) per ion type, as shown in Eq. (1):

1 $${P}_{w}(\%)=\frac{{N}_{{{\rm{Hydrates}}}}}{{N}_{{total}}}\times 100$$

Hydration propensity varies dramatically when calculated per counterion type (Fig. 1c). Data for selected common anions are shown in Fig. 1c, with all data provided in Table S3. Mesylate salts are hydrates in ~20% of cases, whereas triflate salts are only hydrates in <5%, and salts containing tosylate ions show intermediate hydration propensities (Fig. 1c). Ionic charge is a dominant factor in hydration, with salts hydrating more frequently than cocrystals (21% vs. 6%). Yet this binary distinction oversimplifies the trend, with triflate salts forming fewer hydrates than typical uncharged cocrystals. Clearly, the role of charge in hydration is more nuanced than simply charged versus uncharged or even divalent versus monovalent. Salts in the CSD and crystalline material formulations frequently contain inorganic counterions, particularly the halides (29% of all CSD salts), which biases trends (Fig. 2). This bias means that any global average for salt hydration will be skewed by these dominant few counterions; thus, analyzing hydration behavior by counterion type is essential to mitigate data bias.

Analysis of the propensities by counterion type revealed that charge localization within the counterion structure strongly influences the hydration propensity of salts (Fig. 1c). For example, among halides, hydration propensity decreases with increasing ionic radius: iodide salts form roughly half as many hydrates as chloride salts. Similarly, inductive effects reduce hydration propensity: with alkyl halide groups alpha to the oxyanion, withdrawing electron density and lowering hydration propensity. Resonance and intramolecular hydrogen bonding were also observed to play a role, with salicylate salts exhibiting lower hydration propensity than acetate salts.

To quantify the impact of charge localization in counterions on salt hydration, we calculated molecular electrostatic potentials (ESP) of common counterions using electron densities obtained via density functional theory (DFT). The molecular ESP is a scalar field representing the electrostatic potential generated by a molecule in three-dimensional space, determined by its geometry and charge distribution (nuclear charges and electron density). Computationally, ESP is evaluated by first calculating the electron density and then determining the potential experienced by a positive test charge on a chosen molecular surface. This surface typically corresponds to the van der Waals envelope, defined by an isosurface of low electron density (0.002 e au⁻³ in our study). The maximum and minimum ESP values on this surface indicate the points of strongest interaction with negative and positive charges, respectively. In other words, these values quantify the strongest intermolecular interactions a counterion can engage in with a charge or dipole. Our goal was to correlate these interaction strengths with hydration propensity and explore how counterion structure influences ESP and, consequently, hydration behavior.

**Fig. 3:**
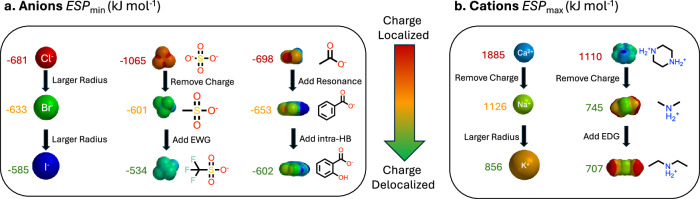
Effects of molecular substituents on charge localization. Influence of counterion structural variations on charge localization on **a** anions and **b** cations. Ions at the top of each column have the highest charge localization, while those below show progressively lower localization. Electrostatic potential (ESP) values are reported in kJ mol⁻¹. Abbreviations: EDG electron-donating group, EWG electron-withdrawing group, intra-HB intramolecular hydrogen bond.

Figure 3 shows ESP maps for representative ions, along with the minimum (for anions) or maximum (for cations) ESP values in kJ mol⁻¹, typically located on the atom bearing the formal charge. We examined how structural factors affect charge localization and ESP values across five ion types. For monoatomic ions, two variables were considered: charge and size. For molecular ions, four variables were explored: charge, electron-withdrawing and electron-donating groups (EWG and EDG), resonance, and intramolecular hydrogen bonding (intra-HB). The progression from highly localized to increasingly delocalized charges is illustrated in Fig. 3.

For monoatomic ions (halides and alkali metals), reducing the formal charge from +2 to +1 (Ca²⁺ → Na⁺) decreases *ESP*_max_ by 725 kJ mol⁻¹ (1885 → 1126 kJ mol⁻¹). Increasing ionic radius also reduces the absolute value of ESP minimum or maximum, as seen in Na⁺ → K⁺ (decrease of 270 kJ mol⁻¹) and Cl⁻ → I⁻ (decrease of 96 kJ mol⁻¹). Thus, charge magnitude has the strongest effect on charge localization in monoatomic ions, followed by ion size. For polyatomic ions (sulfonates, carboxylates, ammoniums), removing charge also reduces ESP but less dramatically than in monoatomic ions. For example, SO₄²⁻ → MeSO₃⁻ lowers |*ESP*_min_| by 461 kJ mol⁻¹, while piperazinium²⁺ → ammonium⁺ lowers |*ESP*_max_| by 265 kJ mol⁻¹. Additional delocalization mechanisms further reduce the absolute value of ESP min or max: resonance with an aromatic ring decreases |*ESP*_min_| by 45 kJ mol⁻¹ (acetate → benzoate), and intramolecular hydrogen bonding reduces |*ESP*_min_| by another 51 kJ mol⁻¹ (benzoate → salicylate). Inductive effects, though smaller, accumulate. Adding fluorine substituents to mesylate reduces |*ESP*_min_| by ~22 kJ mol⁻¹ per substituent, totaling ~67 kJ mol⁻¹ for three of them (mesylate → triflate). For ammoniums, electron-donating groups have a similar effect: replacing a proton with a methyl group reduces |*ESP*_max_| by ~50 kJ mol⁻¹ per substitution (ammonium → dimethylammonium). The length of the alkyl substituent also affects the |*ESP*_max_| with longer alkyl chains stabilizing the charge more effectively.

Overall, counterions with divalent charge (Ca²⁺, piperazinium²⁺, sulfate²⁻) have the highest absolute ESP values (1000–2000 kJ mol⁻¹), followed by monovalent cations (~700–1200 kJ mol⁻¹) and monovalent anions (ESP ~ 500–700 kJ mol⁻¹).

Because |*ESP*_min_| and |*ESP*_max_| reflect the strength of potential intermolecular interactions between counterions and their environment, we investigated whether these descriptors can predict the propensity of salts to hydrate^47–49^. Each salt in our dataset consists of a common counterion paired with an ionized counterpart, meaning each crystal contains both an |*ESP*_min_ | (from the anion) and |*ESP*_max_ | (from the cation). Smaller ions (typically corresponding to the common counterion types identified in Fig. 2) tend to bear the most extreme |*ESP*_min_| and |*ESP*_max_| values. Although our approach considers only one counterion when assessing hydration propensity, this simplification is justified. The common counterion is generally smaller, carries more localized charge, and has more extreme electrostatic potential, making it the primary driver of hydration behavior in molecular salts. Furthermore, when designing salts, the selection is often confined to the choice of counterion. Therefore, a focus on counterion behavior allows for proactive design through a considered screening process.

**Fig. 4:**
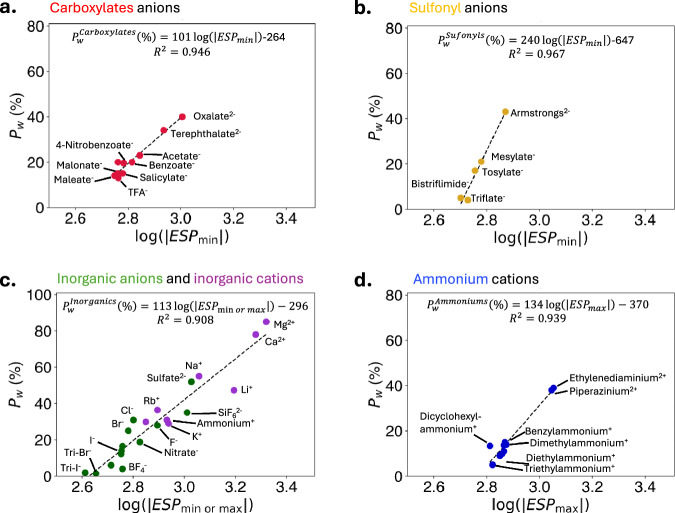
Correlation between electrostatic potential (ESP) extrema and hydration propensity. Relationship between ESP extrema and *P*_*w*_ for salts containing common counterions grouped by family: **a** carboxylate anions, **b** sulfonyl anions, **c** inorganic ions, and **d** ammonium cations. Selected counterions are labeled for illustration; full datasets are provided in Table S3.

Analysis of hydration statistics for molecular salts in the CSD revealed strong linear correlations between the probability of hydration (*P*_*w*_) and the logarithm of the absolute ESP extrema, log|${{ESP}}_{\min }$| or log|${{ESP}}_{\max }$ | , for common counterions (S3). The analysis was further refined by grouping counterions into families that share common geometries and charge distributions (e.g., sulfonates possess three partially charged oxygen atoms in a tetrahedral geometry, whereas carboxylates have two in a planar geometry). Inorganic anions and cations were grouped together, as they show similar behavior. This classification sharpens the overall trends, yielding strong linear correlations (R^2^ > 0.9) between *P*_*w*_ and log|ESP_min or max_| across all families (Fig. 4).

Comparison of the slopes reveals differences between families: sulfonyl anions (240) > ammonium cations (134) > inorganic ions (113) > carboxylates (101). The steeper slope observed for sulfonyl anions might result from the way the charge is distributed. In sulfonates, the ESP minima are present at three sites between the oxygens clustered around the sulfur. These multiple minima are all localized on one side of the ion, with the organic moiety on the other. Like sulfonyl anions, carboxylates also have their charge distributed on one side with an organic moiety on the other. However, because there is only a single ESP minimum located between the two oxygens, fewer interactions are required and result in a lower slope. In contrast, inorganic ions tend to have more uniform charge distributions across their surface, resulting in a greater area that can be interacted with and stabilized since they have no organic moiety. Within each counterion family, *P*_*w*_ decreases as the magnitude of the ESP extrema decreases, regardless of the mechanism of charge delocalization (e.g., ionic charge, ionic radius, electron-withdrawing or donating groups, intramolecular hydrogen bonding, or resonance effects).

The linear relationships identified in Fig. 4a–d allow the hydration propensities for counterions with limited representation in the CSD to be predicted. We compiled a variety of acids and bases together with their acid strengths and predicted salt hydration propensities (Tables 1 and 2). These counterions were then ranked by predicted *P*_*w*_ (${P}_{w}^{{pred}}$). For anions (Table 1), edisylic acid, sulfuric acid, and carbonic acid exhibit the highest predicted hydration propensities (~ 50%), whereas PF_6_^-^, BF_4_^-^, bistriflimide^-^, tribromide^-^, and triiodide^-^ all show much lower hydration propensities (${P}_{w}^{{pred}}$ < 12%). For cations (Table 2), Mg²⁺ and Ca²⁺ are predicted to have the highest hydration propensity (${P}_{w}^{{pred}}$ > 70%), while diisopropylammonium, triethylammonium, and dicylohexylmmonium are among the lowest (*P*_*w*_ < 10%). A more complete table with additional acids and bases with their predicted hydration propensities can be found in tables S1 and S2.

**Table 1:** Collection of acids and their acid strengths (p*K*_a_), together with the corresponding anions, their family classification (fam), number of CSD observations (*N*_CSD_), computed ESP_min_ values, and predicted salt hydration propensities (${P}_{w}^{{pred}}$)

Acid	$p{K}_{a}$ [HA]	Anion	Fam	*N*_CSD_	*ESP*_Min_ (kJ mol^−1^)	${P}_{w}^{{pred}}$(%)
Edisylic acid	−2.2, −1.5^*^	Edisylate^2-^	S	22	−821	52
Sulfuric acid	−3.0, 1.9^*^	Sulfate^2-^	I	434	−1065	46
Carbonic acid	3.8, 10.3^*^	Carbonate^2-^	C	57	−1161	46
H_2_SiF_6_	0.2	SiF_6_^2-^	I	158	−1025	44
Armstrong acid	<1	Armstrong^2-^	S	165	−742	42
Oxalic acid	1.2, 3.5	Oxalate^2-^	C	205	−1013	40
Succinic acid	4.2, 5.6	Succinate^2-^	C	76	−927	36
Fumaric acid	3.0, 4.5	Fumarate^2-^	C	95	−921	35
Terephthalic acid	3.5, 4.3	Terephthalate^2-^	C	133	−861	32
HF	3.2	Fluoride^-^	I	189	−784	31
Acetic acid	4.8	Acetate^-^	C	223	−698	23
Nitric acid	−1.4	Nitrate^-^	I	1099	−669	23
Mesylic acid	−1.9	Mesylate^-^	S	207	−601	20
Benzoic acid	4.2	Benzoate^-^	C	143	−653	20
HCl	−5.9^54^	Chloride^-^	I	7707	−632	20
Nicotinic acid	2.0	Nicotinate^-^	C	37	−638	19
HBr	−8.8^54^	Bromide^-^	I	3791	−603	18
Salicylic acid	3.0	Salicylate^-^	C	148	−602	17
Perchloric acid	−15.2	Perchlorate^-^	I	1722	−572	16
3,5-Dinitrobenzoic acid	2.7	3,5-Dinitrobenzoate^-^	C	162	−577	15
Malonic acid	2.8, 5.7	Malonate^-^	C	103	−575	15
HI	−9.5^54^	Iodide^-^	I	2872	−567	15
Tosylic acid	−1.3	Tosylate^-^	S	511	−569	14
Trifluoroacetic acid	−0.25	TFA^-^	C	369	−566	14
γ-Resorcylic acid	1.1	γ-Resorcylate^-^	C	56	−564	14
Maleic acid	1.9, 6.2	Maleate^-^	C	215	−560	14
Picric acid	0.42	Picrate^-^	CSD	595	−566	12
Triflic acid	−14.7	Triflate^-^	S	1692	−535	12
HPF_6_	−20^55^	PF_6_^-^	I	1415	−518	11
HBr + Br_2_	−8.8^54^	Tribromide^-^	I	130	−450	4
Bistriflimide	−10.2	Bistriflimide^-^	S	207	−503	2
HI + I_2_	−9.5^54^	Triiodide^-^	I	107	−409	~0

The equation used to calculate ${P}_{w}^{{pred}}$ depends on the anion family classification, where C = carboxylates, S = sulfonyl anions, I = inorganic anions, and CSD means the *P*_*w*_ was obtained directly from the CSD. The table is ranked by anions from highest to lowest predicted ${P}_{w}^{{pred}}$. Where multiple acidic groups are present, multiple p*K*_a_ values are reported, with the corresponding ESP and hydration propensity calculated for the specific anion shown.

Note: pK_a_ values were taken from various sources. Unless otherwise stated, pK_a_ values were obtained from the National Institute of Health (NIH). (*) pK_a_ was calculated using ChemAxon’s chemicalize, (^‡^) pk_a_ was taken from DrugBank.

**Table 2:** Collection of bases and the acid strengths of their conjugated protonated base (p*K*_a_), together with the corresponding cations, their family classification (fam), number of CSD observations (*N*_*CSD*_), computed ESP_max_ values, and predicted salt hydration propensities (${P}_{w}^{{pred}}$)

Base	$p{K}_{a}$ [BH + ]	Cation	Fam	*N*_CSD_	*ESP*_Max_ (kJ mol^−1^)	${P}_{w}^{{pred}}$(%)
Mg^2+^(OH^-^)_2_	14.8	Magnesium^2+^	I	266	2092	79
Ca^2+^(OH^-^)_2_	14.8	Calcium^2+^	I	215	1901	75
Na^+^OH^-^	14.8	Sodium^+^	I	804	1141	49
Ethylenediamine	7.5, 10.7	Ethylenediaminium^2+^	A	160	1129	38
Piperazine	5.6, 9.8	Piperazinium^2+^	A	196	1110	37
K^+^OH^-^	14.8	Potassium^+^	I	1017	867	36
Ammonia	9.3	Ammonium^+^	I	799	854	31
Rb^+^OH^-^	14.8	Rb^+^	I	143	784	31
Cs^+^OH^-^	14.8	Cs^+^	I	221	706	26
DMAP	9.6	DMAP^+^	CSD	165	631	25
DABCO	3.0, 8.8	DABCO^2+^	A	76	811	19
Dimethylamine	10.6	Dimethylammonium^+^	A	169	748	15
Imidazole	7.0	Imidazolium^+^	P	142	722	15
Benzylamine	9.8	Benzylammonium^+^	A	127	738	14
Morpholine	8.5	Morpholinium^+^	A	107	734	13
T-butylamine	10.7	T-butylammonium^+^	A	102	732	13
Cyclohexamine	10.6	Cyclohexammonium^+^	A	92	723	13
1-PEA	9.59	1-PEA^+^	A	346	722	12
Trimethylammonia	9.7	Trimethylammonium^+^	A	71	714	12
Pyridine	5.2	Pyridinium^+^	CSD	182	710	12
Piperidine	11.1	Piperidinium^+^	A	94	710	11
Diethylammonia	11.0	Diethylammonium^+^	A	107	707	11
Diisopropylamine	11.1	Diisopropylammonium^+^	A	73	675	9
Triethylamine	10.8	Triethylammonium^+^	A	263	664	8
Dicyclohexylamine	10.4	Dicylohexylammonium^+^	A	149	649	6

The equation used to calculate ${P}_{w}^{{pred}}$ depends on the cation family classification, where I = inorganic anions, A = ammonium cations, and CSD means the *P*_*w*_ was obtained directly from the CSD. The table is ranked by anions from highest to lowest predicted ${P}_{w}^{{pred}}$. Where multiple basic groups are present, multiple p*K*_a_ values are reported for the conjugated bases, with the corresponding ESP and hydration propensity calculated for the specific cation shown (1-PEA: 1-phenylethylamine).

Note: pK_a_ values were taken from various sources. Unless otherwise stated, pK_a_ values were obtained from the National Institute of Health (NIH). In hydroxides, water is the protonated base (with pK_a_ = 14.8) and the metal acts as an observer in the deprotonation and salt formation.

Tables 1 and 2 are a resource to guide salt design. Table 1 compiles acidic compounds together with their corresponding anions, to design salts of basic AMIs (the most common case for pharmaceutical APIs), while Table 2 compiles basic compounds with their corresponding cations, for the design of salts for acidic AMIs. An initial screening for suitable acids or bases can be performed based on p*K*_a_ (ensuring salt formation). Once a list of prospective acids or bases has been identified, considering ${P}_{w}^{{pred}}$ can help to screen a variety of hydration propensities rationally.

**Fig. 5:**
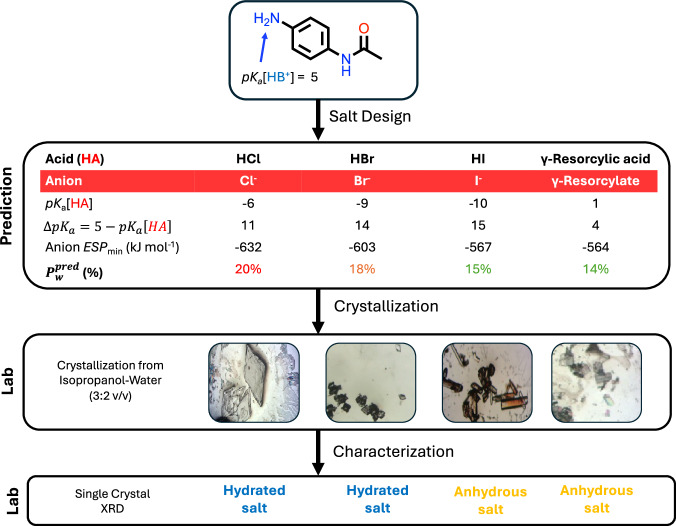
Experimental demonstration of ESP-based hydration probability predictions and their use for salt design. The compound 4-aminoacetanilide (4-AM) is weakly basic with *pK*_a_ of 5, and unknown salt formation behavior. We proceeded to design our salt screening by: **a** using the Δ*pK*_a_ rule to select acids of the correct strength, and **b** by exploring salts leading to different hydration propensities. These predictions informed the choice of acids and the experiments. Crystallization from isopropanol: water (3:2 v/v) yielded single crystals whose structures were confirmed by X-ray diffraction. As anticipated, all pairings led to salt formation. Excitingly, the two salts with the two highest hydration probabilities led to hydrated salts, whilst the two salts with the lowest hydration probabilities led to anhydrous salt.

To illustrate the practical application of our predictive framework, we performed a salt screening experiment on a basic molecule with unknown salt formation behavior: 4-aminoacetanilide (4-AM, Fig. 5). The *pK*_a_ of the protonated compound was estimated to be 5. Four acids were selected, HCl, HBr, HI, and γ-resorcylic acid, based on their availability and required acid strength (p*K*_a_ ≤ 1). 4-AM’s weak basicity limited the number of viable acids, as many carboxylic acids are not strong enough to protonate the primary amine. All choices ensured Δ*pK*_a_ > 4, satisfying the proton transfer criterion for salt formation. Predicted hydration propensities for the corresponding anions were 20% (Cl⁻), 18% (Br⁻), 15% (I⁻), and 14% (γ-resorcylate), enabling rational counterion selection. Crystallization was carried out from isopropanol: water mixtures (3:2 v/v) under controlled cooling conditions, yielding good-quality single crystals for all four salts. Single-crystal X-ray diffraction confirmed salt formation in all cases (Figs. S4–S7) as anticipated by the Δp*K*_a_ rule. Excitingly, the two salts predicted to have the highest hydration propensities (chloride and bromide) crystallized as hydrates, whereas the two salts predicted to have the lowest propensity (iodide and γ-resorcylate) crystallized as anhydrous. While these four salts represent only a portion of every possible salt (and conceivably could have been all anhydrous), it demonstrates how the framework can be applied. If the hydrated bromide salt was obtained first, it would suggest going lower in propensity (as done with iodide and γ-resorcylate) to identify a potential anhydrous salt form.

**Fig. 6:**
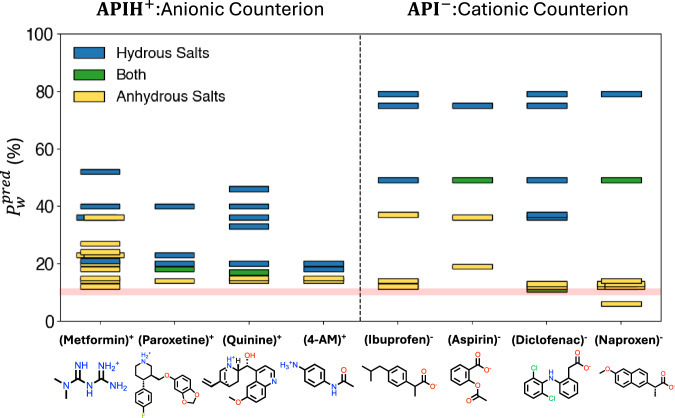
Occurrence of hydrous and anhydrous salts for four basic (left) and four acidic (right) AMIs with multiple reported salts in the CSD. Each bar represents a crystal structure of an AMI paired with a common counterion. For example, quinine salts span from maleate⁻ (lowest yellow bar) to sulfate²⁻ (highest blue bar). Hydrated salts are shown in blue, anhydrous salts in yellow, and counterions exhibiting both hydrated and anhydrous forms in green. 4-AM⁺ refers to salts of 4-aminoacetanilide prepared in the screening experiments. The red line indicates the average ${P}_{w}^{{pred}}$ of the AMIs based on their ESP-derived values and corresponding hydration equations (ammoniums and carboxylates).

To build on our example of 4-AM and further understand the impact of the AMI structure on salt hydration (beyond that of the smaller common counterion from which ${P}_{w}^{{pred}}$ is calculated), we examined hydration trends in salts of eight AMIs (Fig. 6). Alongside 4-AM, we selected three basic and four acidic AMIs that form salts with a range of common counterions. We then evaluated whether the predicted salt hydration propensities ${P}_{w}^{{pred}}$ (calculated from the structure of the common counterion alone), translate into consistent behavior within individual AMI systems.

Across the analyzed examples (Fig. 6), there seems to be a system-dependent hydration threshold in ${P}_{w}^{{pred}}$, denoted ${P}_{w}^{{pred},\,*}[{AMI}]$, above which hydration of salts generally occurs. Below this limit, predominantly anhydrous salts appear, while those above it tend to be hydrous. While the outcome will also depend on crystallization conditions, this observation is useful, as the formation of an anhydrous salt suggests that salts of counterions with lower values of ${P}_{w}^{{pred}}$ are also likely to be anhydrous.

Although exceptions exist and are expected (e.g., the cinnamate salt of metformin), the identified salts follow the ranking of common counterions. While specific interactions between the AMI and the counterion undoubtedly influence hydration outcomes, the relative ordering of salts based on counterion-derived ${P}_{w}^{{pred}}$ remains congruent with the broader CSD trends. These rankings are effective, as rationalized earlier, because common counterions are typically small and possess localized charges, resulting in more extreme ESP values than those of the larger AMIs typically involved in salt formation. For example, the average ${P}_{w}^{{pred}}$ computed using the ESP extrema of the eight AMIs studied in Fig. 6 is approximately 15% (shown as a red band), which is lower than that calculated from the structure of the most common counterions reported in Tables 1 and 2. It’s also worth noting that many of the AMIs in Fig. 6, are relatively simple and small compared to many AMIs. This means that 15% may be an overestimate of the average AMI hydration propensity.

Ions with highly localized charge may be more prone to hydrate formation because they rely on external interactions to stabilize their charge. Strong electrostatic repulsions associated with ionization can be mitigated either internally, through inductive or resonance effects within the counterion, or externally, through solvation. Solvation is a well-established mechanism for stabilizing ionic species, with water being particularly effective due to its high dielectric constant, bidirectional hydrogen bonding ability, and small size^41^. Consequently, when intrinsic stabilization within the counterion and interionic stabilization from the oppositely charged species are insufficient, hydrate formation becomes favorable, as water effectively screens electrostatic repulsion.

While ionization is known to promote hydrate formation, considering intramolecular charge stabilization provides a deeper understanding of how charge distribution governs hydration in molecular salts. This insight enables rational counterion selection. Counterions with greater intrinsic charge stabilization can suppress hydration, whereas those with less stabilized charge favor hydrous forms, as demonstrated for 4-AM.

Although counterion selection offers significant control over hydration propensity, the threshold ${P}_{w}^{{pred},\,*}[{AMI}]$ remains system-dependent and relies on the interaction between the AMI and the counterion. Exceptions are therefore expected. While complete control over crystallization remains an aspiration, modeling charge density in counterions provides a framework for predicting hydration behavior in molecular salts. Future work exploring the interplay between AMI structure and counterion properties may reveal further insights into the hydration of molecular salts.

Molecular salts hydrate more readily than non-ionized crystals, yet the precise role of electrostatics in driving this behavior has remained unclear. By analyzing over 31,000 crystal structures and modeling electron density, we reveal that charge localization governs hydrate formation in molecular salts. This outcome reflects not only the contribution of valence but also inductive effects that shape electron density.

We further demonstrate that an averaged view of salt hydration conceals the substantial variability introduced by counterion choice, even within the same family of ions. Predicted hydration propensities span wide ranges (54–1% for anions and 79–6% for cations), providing considerable flexibility to design salts that are either resistant or deliberately prone to hydration. Combined with the Δp*K*_a_ rule, these insights offer guidance to advance the development of crystalline salts with tailored properties.

Future studies will explore how specific interactions between paired ions influence hydration behavior, refining this framework and enabling greater control over salt design. Our findings provide a predictive basis for counterion selection and have the potential to transform salt design across multiple industries, including pharmaceuticals, where hydration propensity can be anticipated and directed.

## Methods

### CSD searches

All searches of the CSD were carried out using version 5.45 (June 2024) of the CSD through ConQuest, applying the ‘best R-factor’ subset with the following filters enabled: 3D coordinates determined, no errors, single-crystal only, not polymeric, and organics only^50^. By searching within the best R-factor subset, we remove redeterminations while retaining polymorphs of salts and salt hydrates. For all the dataset searches, the allowed elements were restricted to C, N, O, S, B, H/D, P, Si, the alkali and alkaline earth metals, and halides. Within this general search, all suitable counterions (those with no intermolecular hydrogen bond donors and >100 unique entries) were identified using the CSD Python API (Active Programming Interface) using Simplified Molecular Input Line Entry System (SMILES) strings. SMILES representations for all components were extracted for each entry in the subset, and the occurrence of each unique structure was tallied across refcodes. Multiple instances of the same structure within a single refcode (e.g., two acetate anions within the same structure) were treated as a single occurrence. Overall, 71 counterions were identified, 49 of which met the inclusion criteria. Once a counterion was identified by SMILES string, it was searched for in ConQuest within the subset mentioned above. All counterions were searched for by molecular structure alone, except maleate and fumarate, which were also distinguished by text search. Overall, this represents a comprehensive screening of the most common counterions in the organic CSD. Once the 49 counterions were retrieved, they were classified into families. The anions consisted of carboxylates and sulfonyl-containing ions; the cations consisted of 1˚, 2˚, and 3˚ ammoniums, while the inorganics are unique in that they span both charges. Overall, this left 3ions uncategorized (picrate^-^, DMAP^+^, and pyridine^+^) while the remaining 46 belong to larger families of ions. The plot of ESP versus *P*_*w*_ showing all counterions together (including picrate^-^, DMAP^+^, and pyridine^+^) can be found in Figure S3. Not every refcode has retrievable SMILES strings. This means that the number of structures identified by ConQuest and SMILES strings is expected to differ slightly. All refcodes are retrievable directly from the CSD, and the refcodes used in our datasets are available in the repository under the Data Availability section.

### ESP calculations

All ESP charges were calculated in Spartan20 software^51^. The ωB97X-D functional and 6-311 G + G(2df,2p) basis set were used to optimize the ion’s geometry at the ground state first, and then to compute the ESP plot^52^. All ESP plots were calculated on a standard 0.002 e au^−3^ molecular surface, with the resolution set to high. The molecules were initially generated through the input of SMILES strings. Riley et al. found the quality of ESP plots to be dependent on functional selection, but somewhat insensitive to the basis set, provided the basis set had polarization functions on atoms heavier than hydrogen^47^. Mehta and Martin identified the ωB97X-D functional to be one of the best-performing hybrid functionals for calculating electrostatic potentials, and the 6-311 G + G(2df,2p) basis set offers a good balance of computational cost and accuracy^53^. Further information on the calculations can be found in the ESI. Just as Riley et al. had concluded, changing the basis set made little difference to the computed ESP minima and maxima. For example, the ESP minimum for maleate was −559.96 kJ/mol with the 6-311 G + G(2df,2p) basis set and −558.51 kJ/mol using the Def2-TZVPPD basis set, whereas for chloride the values were −632.1 kJ/mol and −633.0 kJ/mol. Further comparisons of the methods are presented in Table S4.

### Crystallization experiments

All reagents were purchased from Tokyo Chemical Industry (TCI) and used as received without further purification. Crystallizations were carried out in a mixed solvent of isopropanol and water (3:2 v/v). For each experiment, the selected acid was combined in a 1:1 molar ratio with 0.1 g (0.67 mmol) of 4-aminoacetanilide. The solvent mixture (isopropanol/water, 3:2 v/v) was then added to each vial, 3 mL for the chloride salt and 4 mL for the bromide, iodide, and γ-resorcylate salts. The vials were capped and placed on a hotplate and heated to 45 °C with 800 RPM stirring for approximately 1 h, until the solutions became clear with a faint pink hue. The stir bars were removed, and the vials were recapped. The hotplate was then set to 22 °C with the vials left undisturbed overnight, during which crystals formed.

### Single crystal X-ray diffraction (SCXRD)

SCXRD was conducted for the structural elucidation of the chloride, bromide, iodide, and γ-resorcylate salt of *4-aminoacetanilinide*. All salts of *4-aminoacetanilinide* were collected at a temperature of 120.0(2) K using MoKα radiation (λ = 0.71073 Å) on a Bruker D8 Venture with a Photon III MM C7 or C14 CPAD detector, IμS-III-microsource, focusing mirrors diffractometer equipped with a Cryostream (Oxford Cryosystems 700) open-flow nitrogen cryostat. All hydrogens on heteroatoms were placed by difference map.

## Supplementary information

Supplementary Information

Transparent Peer Review file

## Data Availability

All data generated in the form of ESP calculations and CSD entries in the datasets, are available from the following link 10.15128/r2h128nd79r. The X-ray crystallographic coordinates for structures reported in this study have been deposited at the Cambridge Crystallographic Data Center (CCDC), under deposition numbers 2500653-2500656. These data can be obtained free of charge from The Cambridge Crystallographic Data Center via www.ccdc.cam.ac.uk/data_request/cif. These data are available upon request to the corresponding author.
