# Supplementary material for: Hydration control in molecular salts through counterion selection
Source: Nat Commun. 2026 Jun 18;17:7710. doi: 10.1038/s41467-026-74560-5 (PMC13434127; doi:10.1038/s41467-026-74560-5)
Supplement: Supplementary file 1 [file 41467_2026_74560_MOESM1_ESM.pdf]

Supplementary Information

Hydration Control in Molecular Salts Through Counterion Selection

Henry A. Holleb,[a] Natalie E. Pridmore,[a] Amy V. Hall,[a] Pablo Martinez-Bulit,[b] and Aurora J. Cruz-Cabeza[a*]

[a]*Department of Chemistry, Durham University, Durham, DH1 3LE, UK*
[b]*The Cambridge Crystallographic Data Centre, 12 Union Road, Cambridge, CB2 1EZ, UK*
*email: aurora.j.cruz-cabeza@durham.ac.uk

Table of Contents

SUPPLEMENTAL INFORMATION 1

1. FULL COUNTERION TABLES 2

2. CSD ANALYSIS 5

2.1 CSD SEARCHES 5
1.2 EXPERIMENTAL P_w 7

2. THE ELECTROSTATIC POTENTIAL 9

3. COUNTERIONS OF THE SAME MOLECULE 11

4. EXPERIMENTAL PROCEDURE 13

4. WATER-COUNTERION INTERACTIONS AND CHARGE DENSITY 17

5. REFERENCES 19

1. Full Counterion Tables

Table S1. Collection of acids and their acid strengths (pK_a), together with the corresponding anions, their family classification (fam), number of CSD observations (N_{CSD}), computed ESP_{min} values, and predicted salt hydration propensities (P_w^{pred}). The equation used to calculate P_w^{pred} depends on the anion family classification, where C = carboxylates, S = sulfonyl anions, I = inorganic anions, and CSD means the P_w was obtained directly from the CSD. The table is ranked by anions from highest to lowest predicted P_w^{pred}. Where multiple acidic groups are present, multiple pK_a values are reported, with the corresponding ESP and hydration propensity calculated for the specific anion shown.

Acid	pK_a [HA]	Anion	Fam	N_{CSD}	ESP_{Min} (kJ mol^{-1})	P_w^{pred}(%)
Edisylic acid	-2.2, -1.5*	Edisylate^{2-}	S	22	-821	52
Sulfuric acid	-3.0, 1.9*	Sulfate^{2-}	I	434	-1065	46
Carbonic acid	3.8, 10.3*	Carbonate^{2-}	C	57	-1161	46
H_2SiF_6	0.2	SiF_6^{2-}	I	158	-1025	44
Armstrong acid	< 1	Armstrong^{2-}	S	165	742	42
Oxalic acid	1.2, 3.5	Oxalate^{2-}	C	205	-1013	40
Succinic acid	4.2, 5.6	Succinate^{2-}	C	76	-927	36
Fumaric acid	3.0, 4.5	Fumarate^{2-}	C	95	-921	35
Glutaric acid	4.4, 5.4	Glutarate^{2-}	C	15	-898	34
Adipic acid	4.4, 5.4	Adipate^{2-}	C	51	-868	33
Terephthalic acid	3.5, 4.3	Terephthalate^{2-}	C	133	-861	32
Pimelic acid	4.5, 5.5	Pimelate^{2-}	C	9	-843	32
Thiodiproprionic acid	3.8, 4.7	Thiodiproprionate^{2-}	C	0	-826	31
HF	3.2	Fluoride^{-}	I	189	-784	31
Pamoic acid	2.7, 2.7	Pamoate^{2-}	C	15	-723	25
Acetic acid	4.8	Acetate^{-}	C	223	-698	23
Propionic acid	4.9	Propionate^{-}	C	11	-691	23
Formic acid	3.8	Formate^{-}	C	95	-691	23
Nitric acid	-1.4	Nitrate^{-}	I	1099	-669	23
Acrylic acid	4.3	Acrylate^{-}	C	2	-678	22
4-Toluic acid	4.4	4-Toluate^{-}	C	41	-656	21
Mesylic acid	-1.9	Mesylate^{-}	S	207	-601	20
Cinnamic acid	4.5	Cinnamate^{-}	C	27	-655	20
Chloroacetic acid	2.9	Chloroacetate^{-}	C	20	-653	20
Benzoic acid	4.2	Benzoate^{-}	C	143	-653	20
HCl	-5.9[1]	Chloride^{-}	I	7707	-632	20
Esylic acid	-1.7	Esylate^{-}	S	15	-596	19
Nicotinic acid	2.0	Nicotinate^{-}	C	37	-638	19
Isonicotinic acid	1.8	Isonicotinate^{-}	C	15	-630	19
HBr	-8.8[1]	Bromide^{-}	I	3791	-603	18
Dichloroacetic acid	1.3	Dichloroacetate^{-}	C	24	-611	17

4-Nitrobenzoic acid	3.4	4-Nitrobenzoate$^-$	C	112	-606	17
Salicylic acid	3.0	Salicylate$^-$	C	148	-602	17
Perchloric acid	-15.2	Perchlorate$^-$	I	1722	-572	16
HBF_4	-4.9	BF_4^-	I	1314	-572	16
Hydroxynaphoic acid	2.8	Hydroxynapthoate$^-$	C	12	-582	15
Trichloroacetic acid	0.51	Trichloroacetate$^-$	C	75	-579	15
3,5-Dinitrobenzoic acid	2.7	3,5-Dinitrobenzoate$^-$	C	162	-577	15
Malonic acid	2.8, 5.7	Malonate$^-$	C	103	-575	15
Camsylic acid	1.2[2]	Camsylate$^-$	S	89	-574	15
Metisulfonic acid	-2.4*	Metisulfate$^-$	S	38	-573	15
HI	-9.5	Iodide$^-$	I	2872	-567	15
Thiocyanic acid	0.9‡	Thiocyanate$^-$	I	156	-565	15
Tosylic acid	-1.3	Tosylate$^-$	S	511	-569	14
Besylic acid	-2.8	Besylate$^-$	S	86	-566	14
Trifluoroacetic acid	-0.25	TFA$^-$	C	369	-566	14
γ-Resorcylic acid	1.1	γ-Resorcylate$^-$	C	56	-564	14
Maleic acid	1.9, 6.2	Maleate$^-$	C	215	-560	14
Napsylic acid	-1.8‡	Napsylate$^-$	S	44	-554	13
Picric acid	0.42	Picrate$^-$	CSD	595	-566	12
Triflic acid	-14.7	Triflate$^-$	S	1692	-535	12
HPF_6	-20[3]	PF_6^-	I	1415	-518	11
$HBr + Br_2$	-8.8[1]	Tribromide$^-$	I	130	-450	4
Bistriflimide	-10.2	Bistriflimide$^-$	S	207	-503	2
$HI + I_2$	-9.5[1]	Triiodide$^-$	I	107	-409	~0

Note: pK_a values were taken from various sources. Unless otherwise stated, pK_a values were obtained from the National Institute of Health (NIH). (*) pK_a was calculated using ChemAxon's chemicalize, (‡) pk_a was taken from DrugBan

Table S2. Collection of bases and the acid strengths of their conjugated protonated base (pK_a), together with the corresponding cations, their family classification (fam), number of CSD observations (N_{CSD}), computed ESP_{max} values, and predicted salt hydration propensities (P_w^{pred}). The equation used to calculate P_w^{pred} depends on the cation family classification, where I = inorganic anions, A = ammonium cations, and CSD means the P_w was obtained directly from the CSD. The table is ranked by anions from highest to lowest predicted P_w^{pred}. Where multiple basic groups are present, multiple pK_a values are reported for the conjugated bases, with the corresponding ESP and hydration propensity calculated for the specific cation shown (1-PEA: 1-phenylethylamine).

Base	pK_a [BH+]	Cation	Fam	N_{CSD}	ESP_{Max} (kJ mol^{-1})	P_w^{pred}(%)
$Mg^{2+}(OH^-)_2$	14.8	Magnesium^{2+}	I	266	2092	79
$Ca^{2+}(OH^-)_2$	14.8	Calcium^{2+}	I	215	1901	75
Li^+OH^-	14.8	Lithium$^+$	I	264	1563	65
Na^+OH^-	14.8	Sodium$^+$	I	804	1141	49
Ethylenediamine	7.5, 10.7	Ethylenediaminium^{2+}	A	160	1129	38
Piperazine	5.6, 9.8	Piperazinium^{2+}	A	196	1110	37
K^+OH^-	14.8	Potassium$^+$	I	1017	867	36
Ammonia	9.3	Ammonium$^+$	I	799	854	31
Rb^+OH^-	14.8	Rb^+	I	143	784	31
Cs^+OH^-	14.8	Cs^+	I	221	706	26
DMAP	9.6	DMAP$^+$	CSD	165	25	25
DABCO	3.0, 8.8	DABCO^{2+}	A	76	811	19
Aniline	4.6	Anilinium$^+$	A	52	754	15
Dimethylamine	10.6	Dimethylammonium$^+$	A	169	748	15
Imidazole	7.0	Imidazolium$^+$	P	142	722	15
Benzylamine	9.8	Benzylammonium$^+$	A	127	738	14
Morpholine	8.5	Morpholinium$^+$	A	107	734	13
T-butylamine	10.7	T-butylammonium$^+$	A	102	732	13
Cyclohexamine	10.6	Cyclohexammonium$^+$	A	92	723	13
1-PEA	9.59	1-PEA$^+$	A	346	722	12
Trimethylammonia	9.7	Trimethylammonium$^+$	A	71	714	12
Pyridine	5.2	Pyridinium$^+$	CSD	182	710	12
Piperidine	11.1	Piperidinium$^+$	A	94	710	11
Diethylammonia	11.0	Diethylammonium$^+$	A	107	707	11
Diisopropylamine	11.1	Diisopropylammonium$^+$	A	73	675	9
Triethylamine	10.8	Triethylammonium$^+$	A	263	664	8
Dicyclohexylamine	10.4	Dicylohexylammonium$^+$	A	149	649	6

Note: pK_a values were taken from various sources. Unless otherwise stated, pK_a values were obtained from the National Institute of Health (NIH). In hydroxides, water is the protonated base (with pK_a =14.8) and the metal acting as observer in the deprotonation and salt former.

2. CSD Analysis

2.1 CSD Searches

All searches were performed using ConQuest[4,5] (CSD version 5.45, June 2024), within the best R-factor subset with the following filters:

- 3D coordinates determined
- No errors
- Single-crystal only
- Not polymeric
- Organics only

An initial element filter reduced the 746,345 unique entries (excluding redeterminations while retaining polymorphs) in the *best R-factor* subset to 395,494 organic entries.

Counterion Inclusion Criteria

An initial screening for counterions was performed using the CSD Python API. Within the broad subset of 395 494 entries, SMILES strings were retrieved and tallied. If no SMILES strings were extractable, the refcode was skipped. Counterions that were present in at least 100 non-redetermination structures were gathered (71 in total). The limit of ≥100 unique entries was set to maintain statistically meaningful data. Once these counterions were identified, they were searched for by structure using ConQuest with the same filters applied. Fumarate and maleate were also distinguished by word search. It was from these searches that P_w was calculated. Counterions with hydrogen-bond donors not interacting with an oxyanion or acting as the protonated ammonium site were excluded to isolate the effect of substituents on charge localization. Counterions where the charge is inaccessible (for example quaternary ammoniums) were also excluded. These criteria yielded a final dataset of 49 counterions. These counterions were then grouped by families (carboxylates, sulfonyl anions, ammoniums, and inorganics) 46 of the 49 counterions belonged to one of these families. While most common counterions were retained, some useful species (e.g. tartrate) were excluded due to these restrictions. The metals were restricted from having metal-carbon or metal-hydrogen bonds, with their corresponding charge specified (+1 for the alkali and +2 for the alkaline earth metals). For the Carboxylates, Sulfonyl Anions, and Inorganic Anions conjugate bases of carboxylic, sulfonic/sulfonamide acids, and inorganic acids with >100 entries were included. The ammoniums included the most common conjugate acids of 1˚, 2˚, and 3˚ ammonium bases with >100 entries. Alkali and alkaline earth metals and other nitrogen-based inorganic cations with at least 100 entries in the CSD. No limit was placed on the number of components per entry. Consequently, while most entries are salts, the datasets also include some ionic cocrystals. These salts and ionic cocrystals can be hydrates, solvates, or heterosolvates. We prioritized retaining a larger dataset with more examples per counterion rather than applying stricter filters that would reduce both the number of entries and the diversity of counterions included. Entries containing a water molecule (H_2O) were classified as hydrated, including both hydrates and hydrate-heterosolvates. For each counterion, an initial search was performed, followed by a secondary search for water to determine the number of hydrated structures. Waters coordinating metals were also considered hydrates, although the nomenclature for these hydrates traditionally differs.

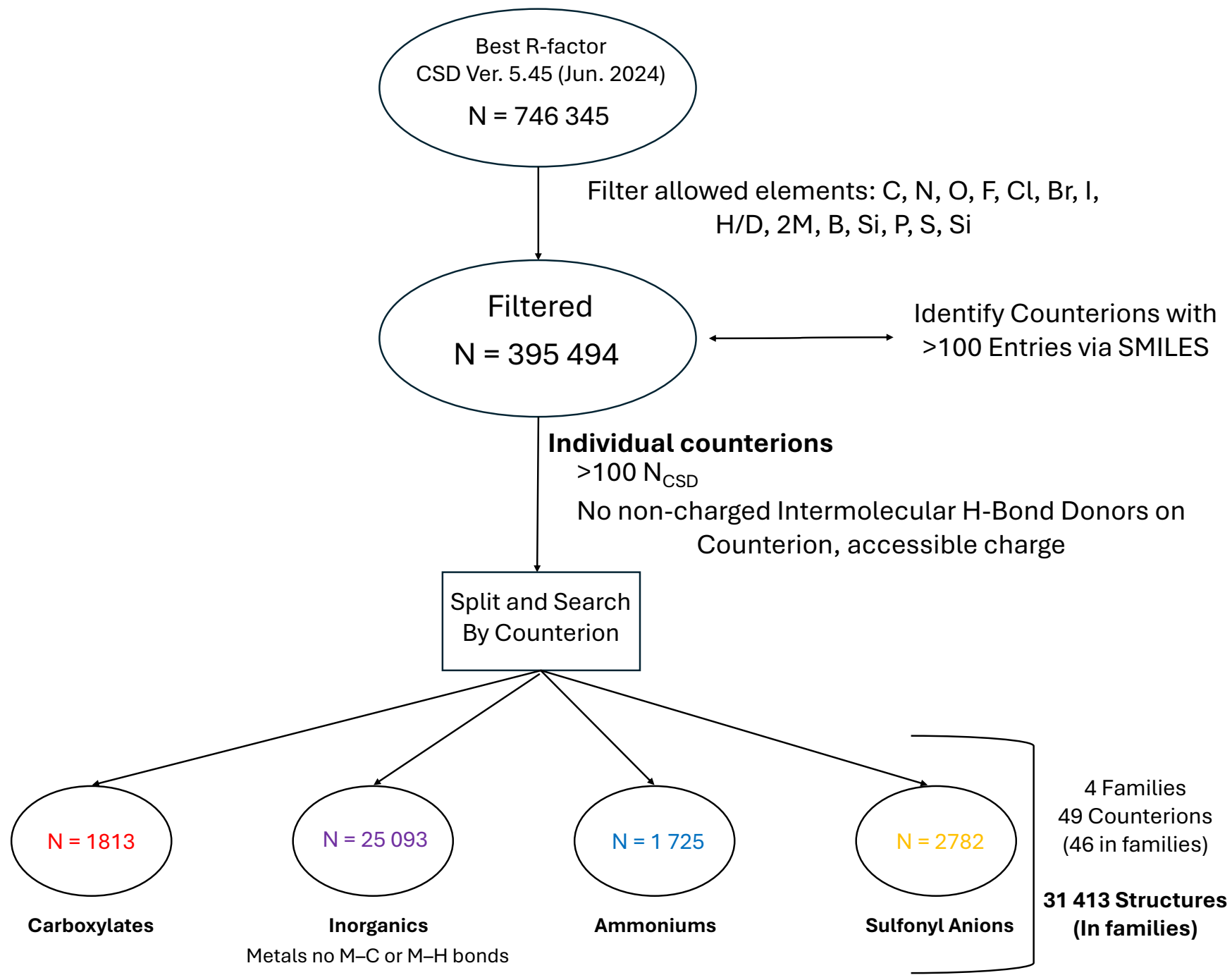

Figure S1. Diagram showing the search criteria for counterion types in the CSD. Once the allowed elements were restricted, a comprehensive screening was conducted via SMILES string to identify counterions with >100 CSD entries. Once identified, these counterions were searched for by structure in ConQuest.

Intermolecular Donors.

When investigating counterions that have two potential protonation states, if the second acidic proton does not form intramolecular hydrogen bonds (e.g. maleate and malonate) or always have intermolecular donors e.g. tartrate or citrate, we noticed two trends. Apart from oxalic acid (oxalate^{2-}: 205, Oxalate^{-}: 264), the divalent form is generally slightly more common (fumarate^{2-}: 95, fumarate^{-}: 84), (succinate^{2-}: 76, succinate^{-}: 68), (terephthalate^{2-}: 133, terephthalate^{-}: 28), (sulfate^{2-}: 434, sulfate^{-}: 292). The same can be observed among cations (piperazine^{2+}: 196, piperazine^{+}: 22), (ethylenediammonium^{+}: 160, ethylenediammonium^{+}: 18). This is consistent with previous work that identified the second proton transfer requires a much smaller ΔpK_a to occur than the first.[6]

The second trend we observe is that counterions that contain intermolecular hydrogen bond donors, either through retaining a second acidic proton or from additional hydroxyl or amine groups, tend to hydrate more than their ESP would predict and in ways that are difficult to rationalize (Figure S2). For this reason, we excluded these counterions. Other important counterions such as citrate^{-} (N_{CSD} = 25), lactate^{-} (N_{CSD} = 8), and malate^{-} (N_{CSD} = 59) were also excluded due an insufficient

number of structures. A plot of the anions, including these additional counterions that retain intermolecular donors highlighted in red, is presented in Figure S2.

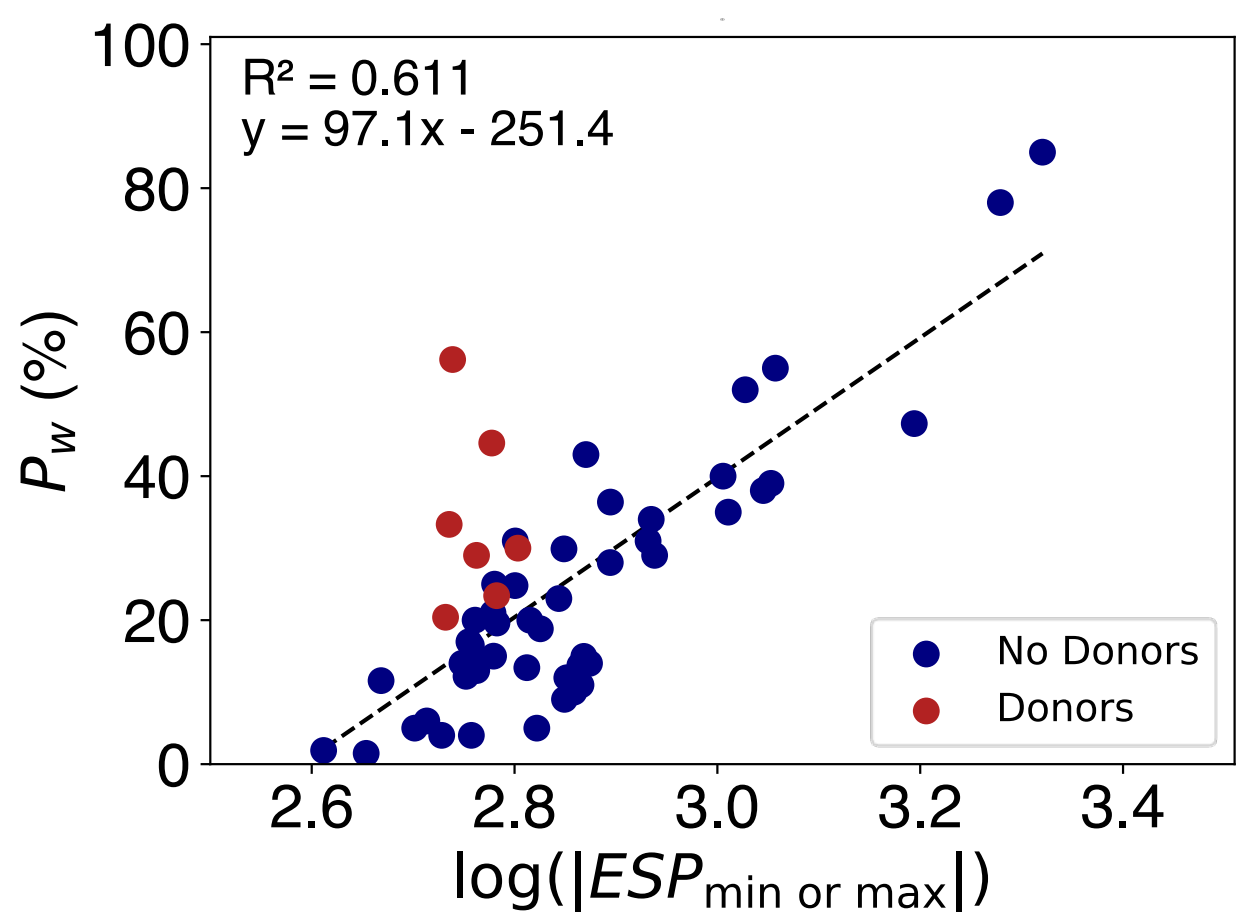

Figure S2. Experimental P_w versus log|$ESP_{\text{min or max}}$| for counterions including anions with intermolecular donors with $N_{CSD} > 100$ plotted in red.

While the hydration probabilities of counterions that do not have intermolecular hydrogen bond donors are generally well predicted by their charge distribution, the ones that do remain more challenging to rationalize.

1.2 Experimental P_w

Table S3. Values P_w calculated from CSD entries with the number of ions N_{total}. P_w CSD is directly calculated from the ratio of hydrates to total structures in the CSD, and N_{CSD} is the total number of entries in the dataset containing that counterion. C = carboxylate, S = sulfonyl anion, I = Inorganic, A = ammonium, O = other

Dataset Ions	Fam	P_w CSD (%)	ESP min or max (kJ mol^{-1})	N_{Total}	$N_{Hydrates}$
Maleate$^-$	C	14	-560	215	31
Malonate$^-$	C	15	-575	103	15
Oxalate^{2-}	C	40	-1013	205	82
Terephthalate^{2-}	C	34	-861	133	45
Acetate$^-$	C	23	-698	223	51
Benzoate$^-$	C	20	-653	143	29
Salicylate$^-$	C	15	-602	148	22
TFA$^-$	C	13	-579	369	48
4-Nitrobenzoate$^-$	C	20	-606	112	22

3,5-Dinitrobenzoate$^-$	C	20	-577	162	33
Tosylate$^-$	S	17	-569	511	88
Mesylate$^-$	S	21	-601	207	43
Triflate$^-$	S	4	-535	1692	65
Bistriflimide$^-$	S	5	-503	207	10
Armstrong^{2-}	S	43	-742	165	71
Picrate$^-$	O	12	-566	595	69
Sulfate^{2-}	I	53	-1065	434	228
Nitrate$^-$	I	19	-626	1099	207
Tribromide$^-$	I	2	-450	130	2
Triiodide$^-$	I	2	-409	107	2
BF_4^-	I	4	-572.	1314	52
PF_6^-	I	6	-518	1415	83
Thiocyanate$^-$	I	12	-565	156	19
Perchlorate$^-$	I	17	-572	1722	285
SiF_6^{2-}	I	35	-1025	158	56
F^-	I	28	-784	189	53
Cl^-	I	31	-632	7707	2392
Br^-	I	25	-603	3791	936
I^-	I	14	-567	2872	405
Cations					
Mg^{2+}	I	85	2092	310	262
Ca^{2+}	I	78	1901	215	168
Li^+	I	47	1564	264	125
Na^+	I	55	1141	887	485
K^+	I	29	867	1160	337
Rb^+	I	36	784	143	52
Cs^+	I	30	706	221	66
Ammonium$^+$	I	31	854	799	247
Pyridinium$^+$	O	12	710	182	21
DMAP$^+$	O	25	632	165	41
Dimethylammonium$^+$	A	14	748	169	23
Ethylenediaminium^{2+}	A	39	1129	160	62
1-PEA$^+$	A	10	722	346	36
Benzylammonium$^+$	A	15	738	127	19
T-butylammonium$^+$	A	14	732	102	14
Diethylammonium$^+$	A	9	707	107	10

Triethylammonium^{+}	A	5	664	263	12
Morpholinium^{+}	A	11	734	107	12
Piperazinium^{2+}	A	38	1110	195	74
Dicyclohexammonium^{+}	A	13	649	149	20

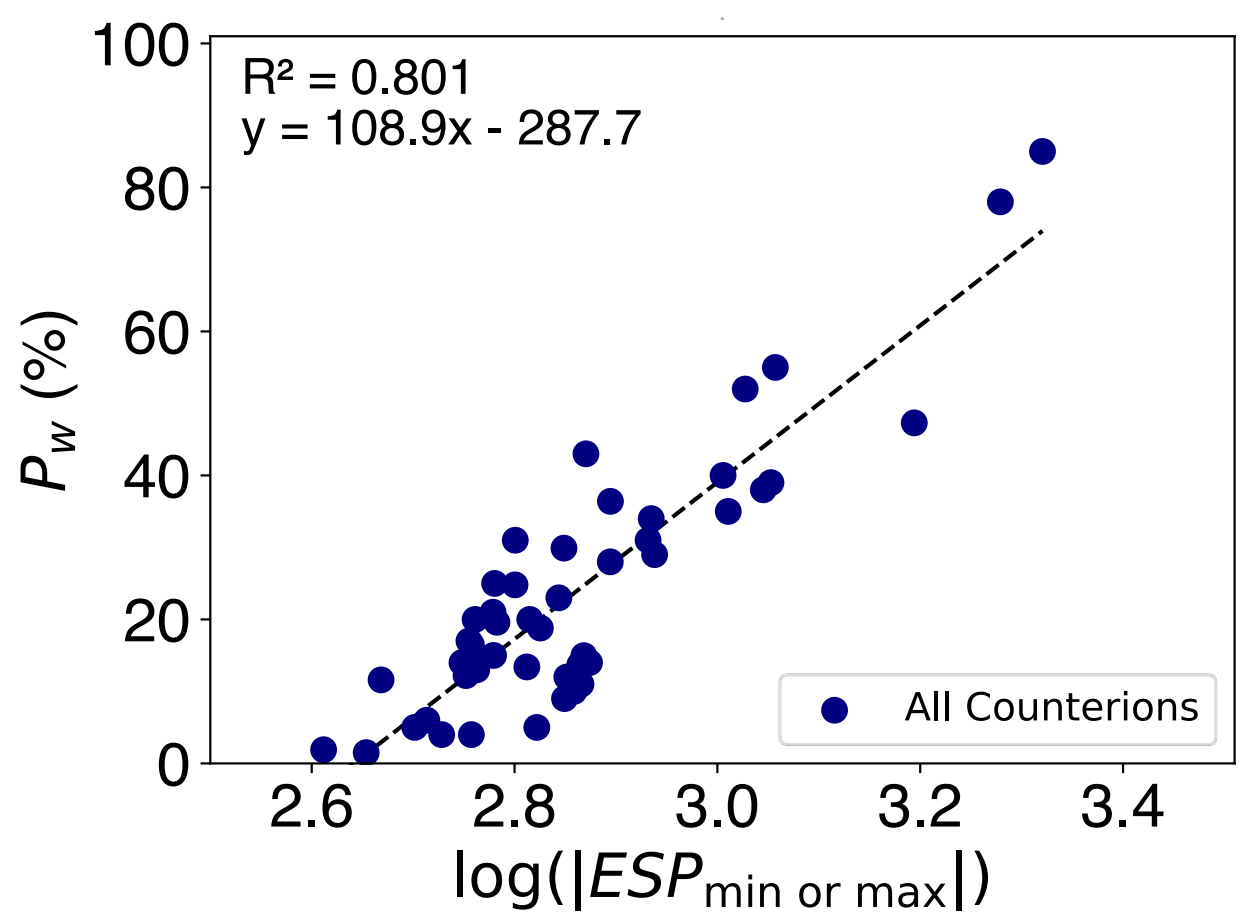

Figure S3. Scatterplot of ESP minimum or maximum vs. P_w for all 49 counterions without hydrogen bond donors and >100 CSD entries.

2. The Electrostatic Potential

Calculating the Electrostatic Potential (ESP)

Electrostatic potentials (ESP) for all counterions were computed using Spartan20.[7] Each ion was generated from its SMILES string and initially minimized using the Merck Molecular Force Field (MMFF)**.** Geometry optimization was then performed at the ground state in the gas phase using the ωB97X-D functional with the 6-311G+G(2df,2p) basis set.[8,9] Different basis sets give very similar ESP minima and maxima, as shown in Table S2. Following optimization, ESP maps were calculated with the same parameters and plotted on a standard isosurface (0.002 e·au^{-3}). The electrostatic potential minimum (for anions) or maximum (for cations) ESP value was extracted from the software. For visualization, color scales were standardized across categories: seven color bands were applied, and global minimum/maximum values were fixed to maintain consistency within each counterion family.

The electrostatic potential describes the potential energy of a positive non-polarizable unit charge at a point in space due to the distribution of nuclei and electrons in a molecule or ion. It is a scalar field that reflects the distribution of a molecules charge. The value of the electrostatic potential at point *r* is given by equation 1.[10]

$$V(r) = \sum_A \frac{Z_A}{|\boldsymbol{R_A} - \boldsymbol{r}|} - \int \frac{\rho(\boldsymbol{r'})d\boldsymbol{r'}}{|\boldsymbol{r'} - \boldsymbol{r}|} \quad [1]$$

Here, R_A denotes the position vector of nucleus A, Z_A is its atomic number (nuclear charge), r is the point in space where the potential is evaluated, and $\rho(r')$ is the electron density at position r'. This means a highly positive ESP value corresponds to the repulsion between the positive test charge and the nuclei of the molecule, and a strongly negative value corresponds to the favorable interaction between the positive charge and the electron density of the molecule. The electrostatic potential $V(r)$ is calculated in atomic units but is commonly reported as energy per mole (kJ mol^{-1}). Among the sulfonates and carboxylates, the ESP_{min} is always found between the oxygens of each respective functional group. The ESP_{max} is found on the hydrogens of the ammoniums, and among the halides and metals the ESP minimum and maximum are generally evenly distributed across the surface with subtle difference of a couple kJ mol^{-1}.

Table S4. Comparing ESP minima and maxima from different basis sets. ESP maxima and minima were computed for several of the counterions to assess how different basis sets affect the identified electrostatic minimum or maximum. All calculations used the same functional, ωB97X-D**.**

Counterion	ESP min or max<br>6-311G+G(2df,2p) (kJ mol^{-1})	ESP min or max<br>Def2-TZVPPD (kJ mol^{-1})
Maleate$^-$	-559.96	-558.51
Malonate$^-$	-574.78	-573.08
Oxalate^{2-}	-1012.96	-1005.75
Terephthalate^{2-}	-860.50	-856.60
Acetate$^-$	-697.70	-690.98
Benzoate$^-$	-653.19	-648.81
Salicylate$^-$	-601.61	-598.32
TFA$^-$	-579.04	-575.97
γ-Resorcylate$^-$	-564.10	-560.82
Ammonium$^+$	854.43	845.21
Dimethylammonium^{2+}	748.01	744.51
Ethylenediamine^{2+}	1129.33	1127.42
Pyridnium$^+$	710.44	710.45
Diethylammonium$^+$	706.71	711.52
Triethylammonium$^+$	663.67	654.09
Piperazinium^{2+}	1109.90	1109.52
Chloride$^-$	632.10	632.99
Bromide$^-$	603.42	594.10

3. Counterions of the same molecule

Common molecules that form multiple salts were investigated to assess whether the trends observed in the CSD generally applied to individual systems.

Table S5. Salts of AMIs split by counterions. The predicted P_w of the counterion and hydration state is reported for each structure. Both the counterion and main component were searched for with the charge specified. Polymorphic refcodes are listed in this table, however only one bar was placed in figure 5. In cases of both hydrous and anhydrous forms, the hydrate is placed below.

AMI	Counterion	Hydration State	Refcode
Metformin^{+}	Sulfate^{2-}	Hydrate	PAJCAF01
	Oxalate^{2-}	Hydrate	PAJBUY
	Succinate^{2-}	Anhydrate	IMIHIY01
	Fumarate^{2-}	Hydrate	EGOHAN
	Chloride^{-}	Anhydrate	JAMRIY02<br>JAMRIY03
	Acetate^{-}	Anhydrate	OJOSUC02
	Formate^{-}	Anhydrate	JEDGIM
	Nitrate^{-}	Anhydrate	HUZLUJ<br>SAFQIB
	Bromide^{-}	Anhydrate	ETIVUY
	Cinnamate^{-}	Hydrate	EGOHER
	Malonate^{-}	Anhydrate	DELKAK
	Maleate^{-}	Anhydrate	DELJUD
	Picrate^{-}	Anhydrate	NAKWAB
Paroxetine^{+}	Oxalate^{2-}	Hydrate	DIZSEN
	Chloride^{-}	Hydrate	GODVAW
	Bromide^{-}	Both	TUZFOL<br>TUZFIF
	Nitrate^{-}	Hydrate	ALGUZ
	Maleate^{-}	Anhydrate	DIZSIR
Quinine^{+}	Sulfate^{2-}	Hydrate	ZZZMNO01<br>ZZZNKI01
	Oxalate^{2-}	Hydrate	VAMGEZ
	Fumarate^{2-}	Hydrate	VAMGID
	Adipate^{2-}	Hydrate	VAMFUO
	Chloride^{-}	Hydrate	EZOSUK<br>HUBBOX<br>SUDWIX<br>WEYDIN
	Salicylate^{-}	Both	ABITEO<br>WANTOU
	Malonate^{-}	Anhydrate	VAMFOI
	γ-Resorcylate^{-}	Anhydrate	VAMFIC

4-am^{+}	$Chloride^{-}$	Hydrate	-
	$Bromide^{-}$	Hydrate	-
	$Iodide^{-}$	Anhydrate	-
	γ-$Resorcylate^{-}$	Anhydrate	-
$Ibuprofen^{-}$	Mg^{2+}	Hydrate	FUYCOS01
	Ca^{2+}	Hydrate	ISOCEB
	Na^{+}	Hydrate	KASVEG<br>KATNOJ
	$Piperazinium^{2+}$	Anhydrate	LEJGAM
	$Benzylammonium^{+}$	Anhydrate	VUCHUX
	1-PEA^{+}	Anhydrate	VAVEK01<br>VUCHIL<br>VUCHOR01<br>ZATFEJ
$Aspirin^{-}$	Ca^{2+}	Hydrate	BEVWIJ
	Na^{+}	Both	PIKYUG<br>PIKYOA<br>PIKZAN
	K^{+}	Anhydrate	NINFUN
	$DABCO^{2+}$	Anhydrate	NUKXOH
$Diclofenac^{-}$	Mg^{2+}	Hydrate	GOLPIG
	Ca^{2+}	Hydrate	GICCAZ
	Na^{+}	Hydrate	AKOTAV<br>LAHBAB<br>LIQFUN<br>LIQFUN01
	$Piperazine^{2+}$	Hydrate	NIFGIS
	K^{+}	Hydrate	TIBWEH
	$T\text{-butylammonium}^{+}$	Anhydrate	TEQCIE
	1-PEA^{+}	Anhydrate	VAKVIO
	$Diethylammonium^{+}$	Both	RORQEU<br>QIZJUE
$Naproxen^{-}$	Mg^{2+}	Hydrate	ANOMEW<br>ANOMIA
	Na^{+}	Both	ASUBUL<br>HEGFUW<br>IVIDEW<br>KINVEK<br>YOHKOX
	$Benzylammonium^{+}$	Anhydrate	FIWZOC
	$Cyclohexamonnium^{+}$	Anhydrate	FIWYUH
	1-PEA^{+}	Anhydrate	BAQFOS<br>HIFRIX

	Pyridine$^+$	Anhydrate	GIPTIJ
	Dibenzylammonium$^+$	Anhydrate	FIWZUI

4. Experimental Procedure

Crystallizations. All reagents were purchased from Tokyo Chemical Industries (TCI) and used as received without further purification. 0.1 g of 4-aminoacetanilinide *(*4AM*)* was weighed and added to 4 glass vials. One molar equivalent of each acid was then added: 55 µl (12M) HCl, 75 µl (8.8M) HBr, 70 µl HI (9.4M), 0.1 g γ-resorcylic acid. A stock solution of isopropanol water (3:2 v/v) was then added to each vial, 3 ml in the vial with chloride, and 4 ml in the other remaining vials. Adding 4 ml of the stock solution to the chloride vial fully dissolved the mixture. Stir bars were added to each vial which were then placed on a hotplate set at 45 ˚C and left stirring at 800 RPM for about an hour. During this time, the solutions turned clear with a pinkish hue. The stir bars were removed, and the vials were re-capped. The hotplate was then set to 22 ˚C and with the vials left undisturbed overnight, when crystals formed.

Crystallography. The X-ray single crystal data of 4AM.Cl, 4AM.Br, 4AM.I and 4AM. γ Resorcylic acid have been collected at a temperature of 120.0(2) K using MoKα radiation (λ =0.71073Å) on a Bruker D8 Venture with a Photon III MM C7 or C14 CPAD detector, IµS-III-microsource, focusing mirrors diffractometer equipped with a Cryostream (Oxford Cryosystems 700+) open-flow nitrogen cryostat. Using APEX5 software[11], intensities were integrated in SAINTS[12] and absorption corrections based on equivalent reflections were applied using SADABS.[13] The structure was solved using ShelXT[14] structure solution program using Intrinsic Phasing and refined by full matrix least squares against F^2 in ShelXL[15] using Olex2.[16] All non-hydrogen atoms were refined with anisotropic displacement parameters. The hydrogen atom on hetero atoms were found by difference map after all other atoms had been refined, all other hydrogens were located geometrically and refined using a riding model. The 4AM.Br structure is partially disordered and has been modelled with the methyl hydrogens in two positions (AFIX 127). Crystal structure images can be seen in figures S4-7 and crystal data and parameters of refinement are listed in Table S5. Crystallographic data for the structures has been deposited with the Cambridge Crystallographic Data Centre with deposition number CCDC-2500653 to 2500656. Copies of the data can be obtained free of charge on application to CCDC, 12 Union Road, Cambridge CB2 1EZ, UK [fax(+44) 1223 336033, e-mail: deposit@ccdc.cam.ac.uk].

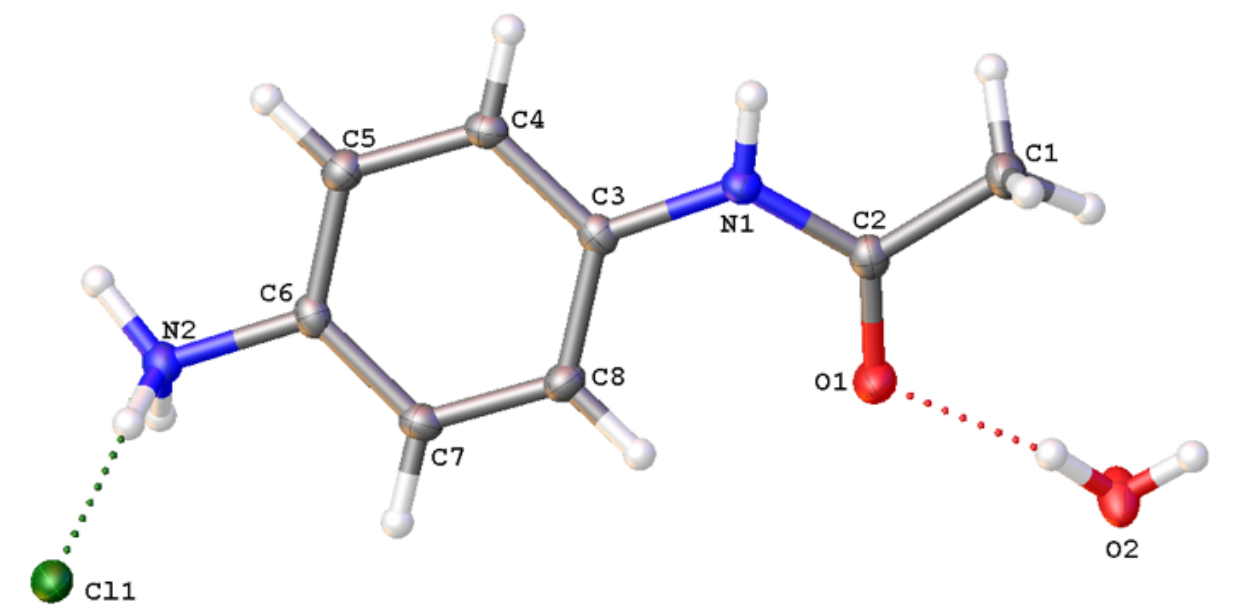

Figure S4. Crystal structure of 4AM.Cl, with the anisotropic displacement parameters depicted at the 50% probability level.

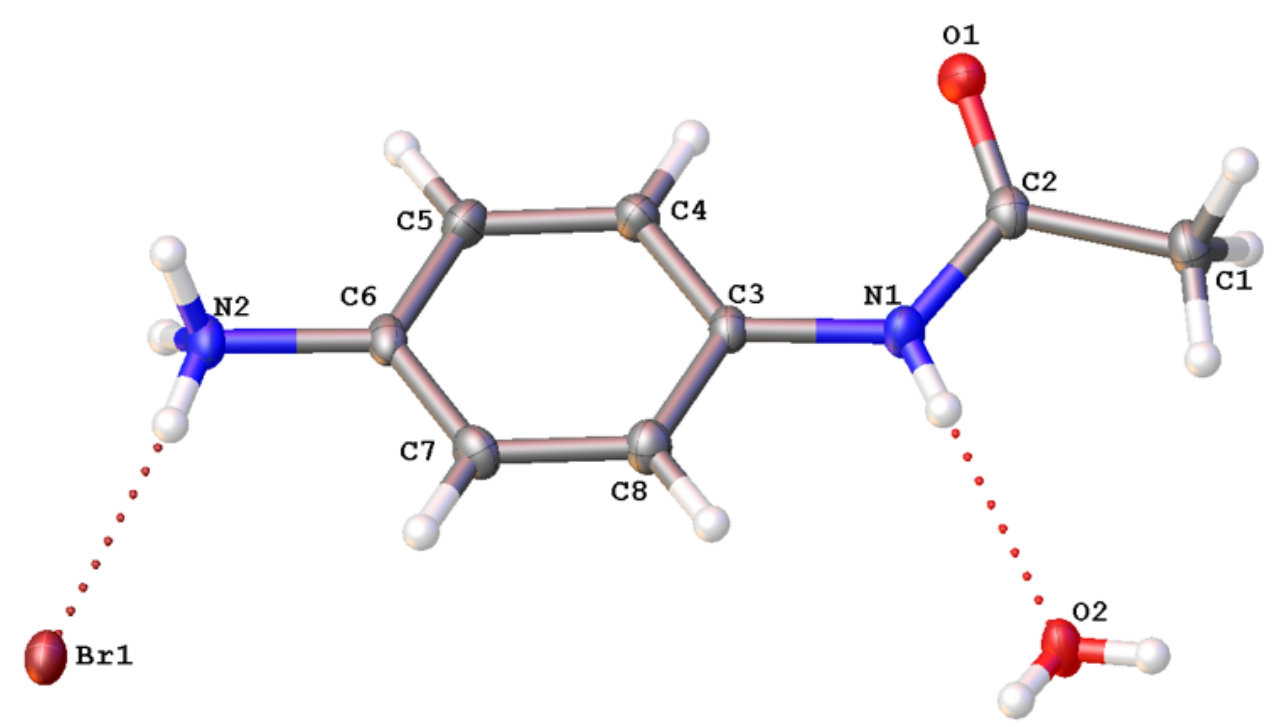

Figure S5. Crystal structure of 4AM.Br, with the anisotropic displacement parameters depicted at the 50% probability level and disorder hidden for clarity.

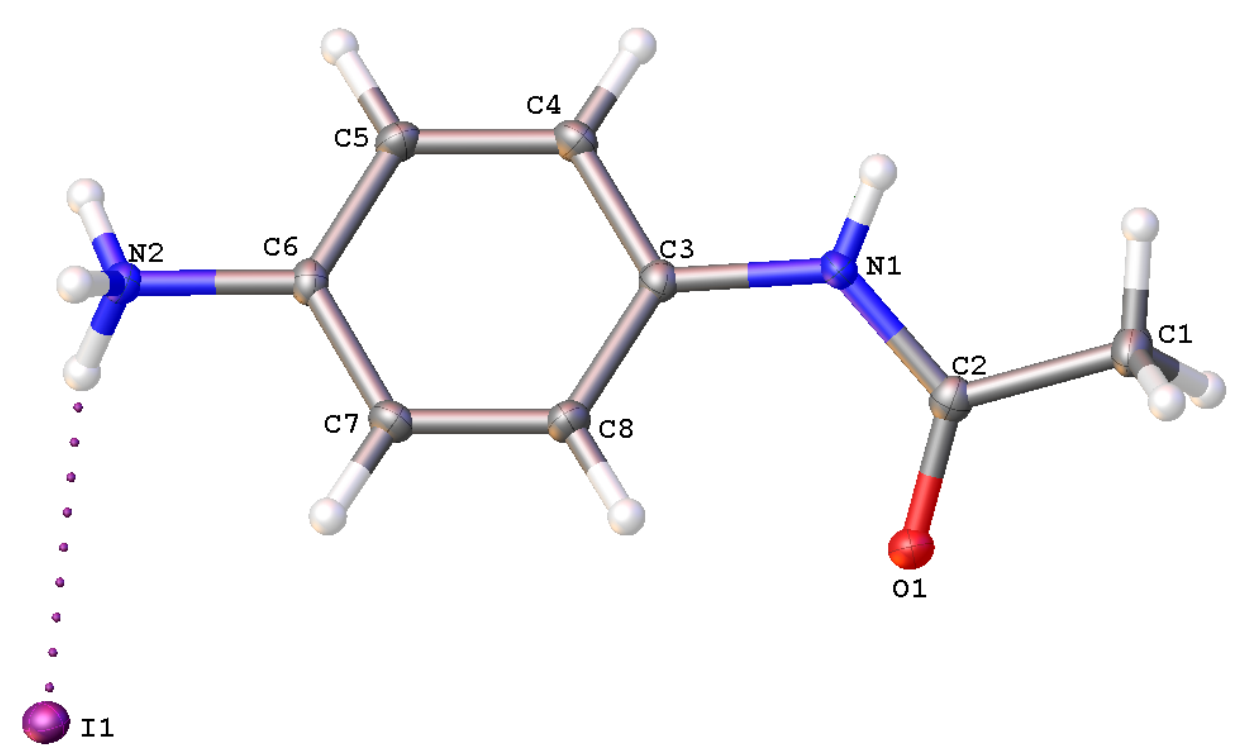

Figure S6. Crystal structure of 4AM.I, with the anisotropic displacement parameters depicted at the 50% probability level.

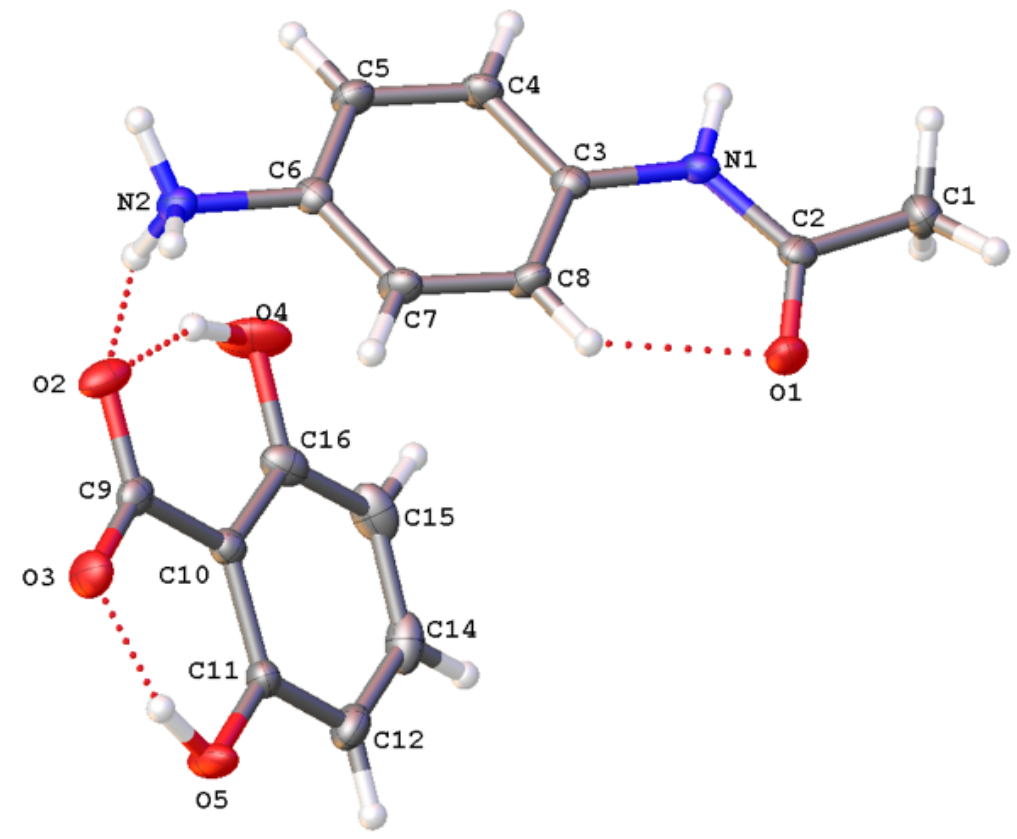

Figure S7. Crystal structure of 4AM. γ-Resorcylate with the anisotropic displacement parameters depicted at the 50% probability level.

Table S6. Crystal data and structure refinement details for 4AM salts.

Compound	4AM.Cl	4AM.Br	4AM.I	4AM.γ-Resorcylate
CCDC number	2500653	2500654	2500655	2500656
Empirical formula	$C_8H_{13}ClN_2O_2$	$C_8H_{13}BrN_2O_2$	$C_8H_{11}IN_2O$	$C_{15}H_{16}N_2O_5$
Formula weight	204.65	249.11	278.09	304.30
Temperature/K	120.00	120.00	120.00	120.00
Crystal system	monoclinic	monoclinic	monoclinic	monoclinic
Space group	$P2_1/c$	$P2_1/c$	$P2_1/c$	$P2_1/c$
a/Å	11.4205(5)	11.4153(4)	5.03160(10)	11.024(4)
b/Å	12.5616(6)	13.0671(4)	17.8858(4)	12.883(4)
c/Å	6.7912(3)	6.7960(2)	10.7249(2)	10.457(4)
α/°	90	90	90	90
β/°	98.2570(10)	96.9010(10)	93.4370(10)	103.060(14)
γ/°	90	90	90	90
Volume/Å^3	964.16(8)	1006.38(6)	963.44(3)	1446.7(9)
Z	4	4	4	4
ρ_{calc}g/cm^3	1.410	1.644	1.917	1.397
μ/mm^{-1}	0.366	4.056	3.280	0.106
F(000)	432.0	504.0	536.0	640.0
Crystal size/mm^3	0.57 × 0.237 × 0.076	0.514 × 0.333 × 0.12	0.221 × 0.101 × 0.073	0.255 × 0.118 × 0.087
Radiation	MoKα (λ = 0.71073)	MoKα (λ = 0.71073)	MoKα (λ = 0.71073)	MoKα (λ = 0.71073)
2Θ range for data collection/°	4.848 to 55.726	4.758 to 56.696	4.434 to 55.752	4.938 to 55.746
Index ranges	$-15 \leq h \leq 15$, $-16 \leq k \leq 16$, $-8 \leq l \leq 8$	$-15 \leq h \leq 15$, $-17 \leq k \leq 17$, $-9 \leq l \leq 9$	$-6 \leq h \leq 6$, $-23 \leq k \leq 23$, $-14 \leq l \leq 14$	$-14 \leq h \leq 14$, $-14 \leq k \leq 16$, $-13 \leq l \leq 13$
Reflections collected	29372	27562	30996	36051
Independent reflections	2295 [R_{int} = 0.0610, R_{sigma} = 0.0277]	2517 [R_{int} = 0.0512, R_{sigma} = 0.0266]	2297 [R_{int} = 0.0486, R_{sigma} = 0.0200]	3450 [R_{int} = 0.0614, R_{sigma} = 0.0270]
Data/restraints /parameters	2295/0/143	2517/0/147	2297/0/126	3450/0/224
Goodness-of-fit on F^2	1.185	1.068	1.086	1.149
Final R indexes [I>=2σ (I)]	R_1 = 0.0367, wR_2 = 0.0846	R_1 = 0.0205, wR_2 = 0.0511	R_1 = 0.0141, wR_2 = 0.0336	R_1 = 0.0515, wR_2 = 0.1058
Final R indexes [all data]	R_1 = 0.0442, wR_2 = 0.0873	R_1 = 0.0242, wR_2 = 0.0526	R_1 = 0.0156, wR_2 = 0.0340	R_1 = 0.0595, wR_2 = 0.1096
Largest diff. peak/hole / e Å^{-3}	0.27/-0.28	0.41/-0.37	0.33/-0.47	0.26/-0.30

4. Water-Counterion Interactions and Charge Density

Water Interactions with Halides. To assess the percentage of structures where water interacts with the halide counterion, the hydrous halide datasets were exported to mercury where the motif search feature was used. The defined motif was water molecules within the sum of the ionic radius of the halide and the Van der Waals radius of oxygen with an O-H…X^- bond angle of 110-180˚.

Water…O^- distances. We evaluated whether more charge dense anions tend to form closer contacts with water. This was accomplished by searching for hydrogen bonds from water O-H…O of the accepting anion using the same search criteria as in the search for water-halide interactions. In the case of carboxylates and sulfonates each oxygen was treated as equivalent (bond distances to the C/S=O and C/S-O^- were both considered). The distributions are presented in Figure S9. Overall, we see no discernable trend between charge density and bond distance and all distances fall within expected strong O-H…O bond distances.

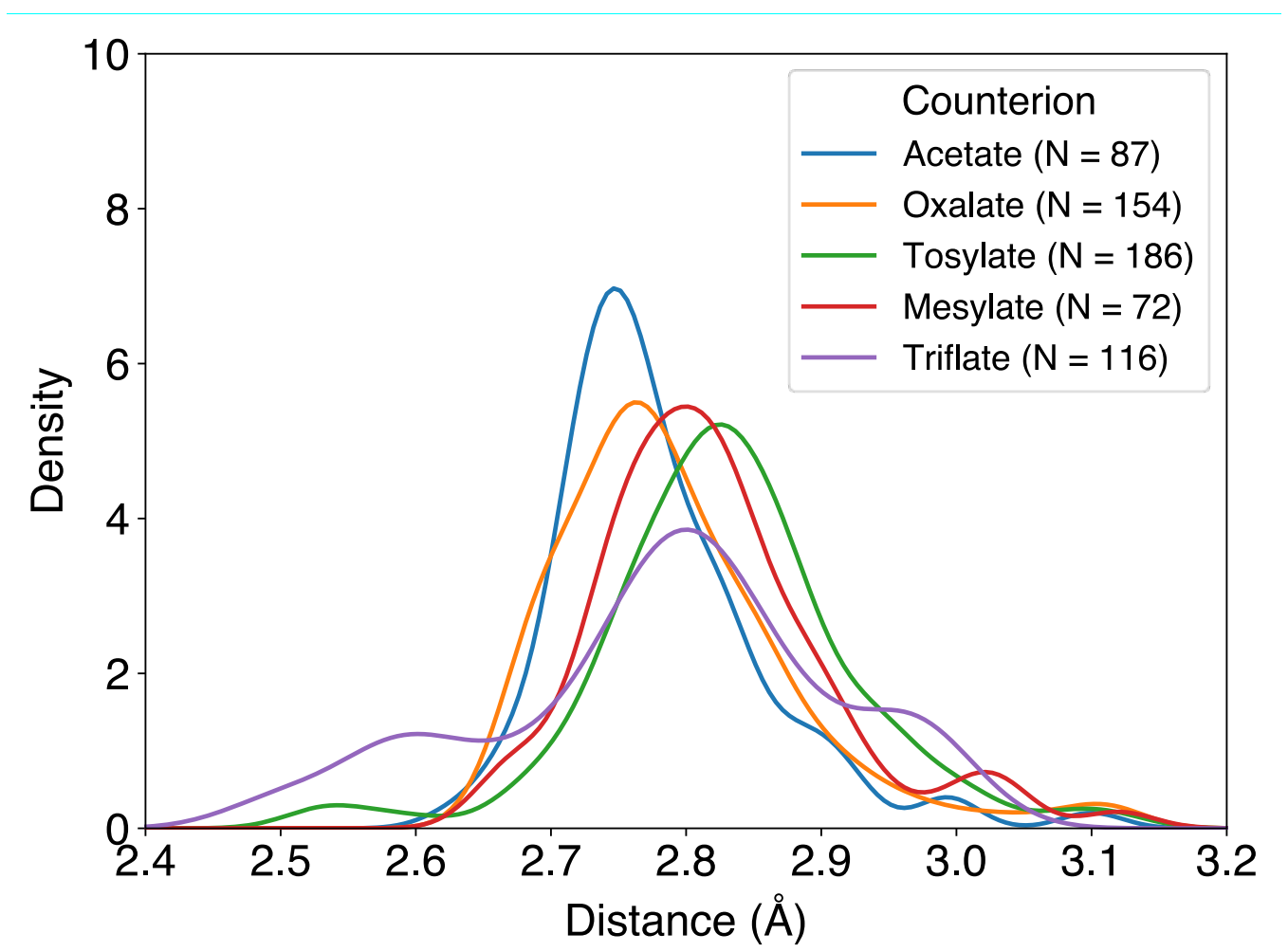

Figure S9. Distribution of water-oxyanion distances in the acetate, mesylate, tosylate, oxalate, and triflate datasets. The number represents the number of interactions included (a structure with three water-O^- contacts will contribute 3).

P_w and Charge Density.

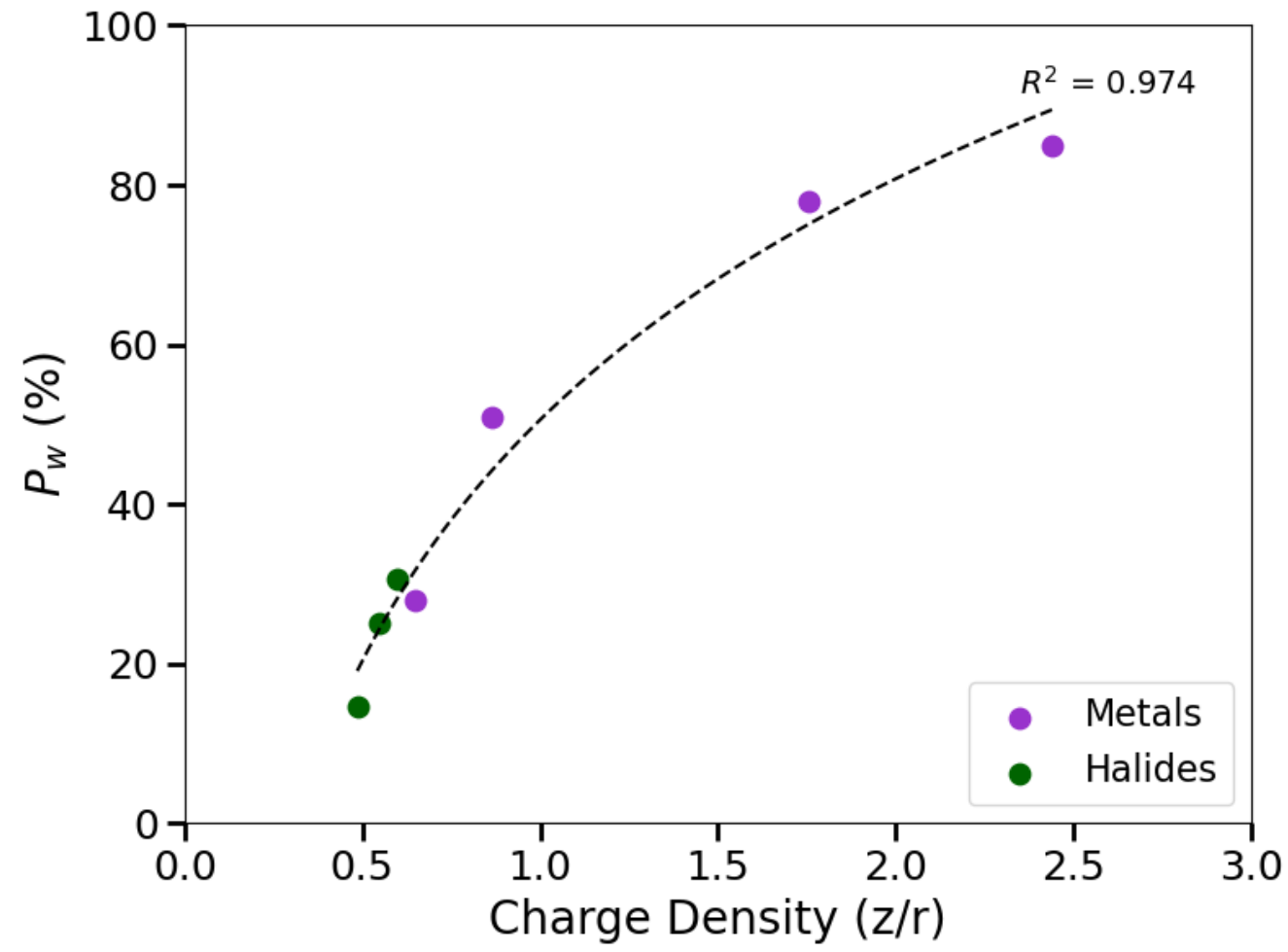

Figure S10. Charge density versus P_w. The charge density (formal charge divided by the ionic radius) of inorganic ions shows a logarithmic relationship with the probability of crystallizing as a hydrate. Charge densities are known to be correlated with hydration enthalpies but had not previously been correlated with crystallization outcomes.

5. References

1. Trummal, A., Lipping, L., Kaljurand, I., Koppel, I. A. & Leito, I. Acidity of Strong Acids in Water and Dimethyl Sulfoxide. *J. Phys. Chem. A* **120**, 3663–3669 (2016).
2. Wang, X. *et al.* Fridel-Crafts Alkylation of Arenes with Indolyl Alcohols for Construction of 3,3-Disubstituted Oxindoles.
3. Miyashita, T., Yasuda, K. & Uda, T. Kinetics and mechanism of hydrolysis of PF_6^- accelerated by H^+ or Al^{3+} in aqueous solution. *Environ. Sci. Water Res. Technol.* **11**, 281–292 (2025).
4. Bruno, I. J. *et al.* New software for searching the Cambridge Structural Database and visualizing crystal structures. *Acta Crystallogr. B* **58**, 389–397 (2002).
5. Groom, C. R., Bruno, I. J., Lightfoot, M. P. & Ward, S. C. The Cambridge Structural Database. *Acta Crystallogr. Sect. B Struct. Sci. Cryst. Eng. Mater.* **72**, 171–179 (2016).
6. Cruz-Cabeza, A. J., Lusi, M., Wheatcroft, H. P. & Bond, A. D. The role of solvation in proton transfer reactions: implications for predicting salt/co-crystal formation using the ΔpKa rule. *Faraday Discuss.* **235**, 446–466 (2022).
7. Shao, Y. *et al.* Advances in methods and algorithms in a modern quantum chemistry program package. *Phys. Chem. Chem. Phys.* **8**, 3172–3191 (2006).
8. Halgren, T. A. Merck molecular force field. I. Basis, form, scope, parameterization, and performance of MMFF94. *J. Comput. Chem.* **17**, 490–519 (1996).
9. Chai, J.-D. & Head-Gordon, M. Long-range corrected hybrid density functionals with damped atom–atom dispersion corrections. *Phys. Chem. Chem. Phys.* **10**, 6615–6620 (2008).
10. Riley, K. E., Tran, K.-A., Lane, P., Murray, J. S. & Politzer, P. Comparative analysis of electrostatic potential maxima and minima on molecular surfaces, as determined by three methods and a variety of basis sets. *J. Comput. Sci.* **17**, 273–284 (2016).
11. APEX 5 v.2023.9-2, Bruker AXS, 2019.

12. Bruker, SAINT+ v8.40.0 Integration Engine, Data Reduction Software, Bruker Analytical X-ray Instruments Inc., Madison, Wisconsin, USA, 2019.

13. Bruker SADABS 2019, Bruker AXS area detector scaling and absorption correction, Bruker Analytical X-ray Instruments Inc., Madison, Wisconsin, USA, 2019.

14. Sheldrick, G. M. *SHELXT* – Integrated space-group and crystal-structure determination. *Acta Crystallogr. Sect. Found. Adv.* **71**, 3–8 (2015).

15. Sheldrick, G. M. Crystal structure refinement with *SHELXL*. *Acta Crystallogr. Sect. C Struct. Chem.* **71**, 3–8 (2015).

16. Dolomanov, O. V., Bourhis, L. J., Gildea, R. J., Howard, J. A. K. & Puschmann, H. *OLEX2* : a complete structure solution, refinement and analysis program. *J. Appl. Crystallogr.* **42**, 339–341 (2009).
