# Supplementary material for: Hydration control in molecular salts through counterion selection
Source: Nat Commun. 2026 Jun 18;17:7710. doi: 10.1038/s41467-026-74560-5 (PMC13434127; doi:10.1038/s41467-026-74560-5)
Supplement: Supplementary file 2 [file 41467_2026_74560_MOESM2_ESM.pdf]

nature portfolio

Peer Review File

Hydration Control in Molecular Salts Through Counterion Selection

Corresponding Author: Professor Aurora Cruz-Cabeza

This file contains all reviewer reports in order by version, followed by all author rebuttals in order by version.

Version 0:

Reviewer comments:

Reviewer #1

(Remarks to the Author)
General comment
This manuscript tackles an important and timely question concerning the hydration tendency of molecular salts. However, the analysis is largely confined to empirical correlations between hydration probability of counterions and their charge density (as quantified by ESP(max/min)), which limits the mechanistic depth of the conclusions.

Major issues
Because water does not distinguish between the API ion and the counterion, considering counterions alone to predict hydration propensity is not justified. Although the hydration propensity of API ions cannot be quantified in the same manner as that of counterions due to the limited number of available salts, this limitation does not justify dismissing the role of the API ion in hydration. Rather, it reflects a constraint of the methodology employed in the present study.
Mechanistic understanding requires treating the ion pair as an integrated entity rather than considering the counterion in isolation. This may help explain why the trends observed for halide salts differ markedly from those of other salts. The current analysis does not capture such ion-pair–specific effects.
CSD may not provide comprehensive data for attaining accurate hydrate propensity for the following reasons:
1. The stability of hydrates depends on a) temperature and b) water activity. Since majority of crystallization experiments were conducted at or near room temperature and many crystallization experiments were performed below water activity of 1, many hydrates are likely missed from the CSD.
2. Many observed hydrates do not have crystal structures solved and, hence, unavailable in CSD for analysis.
3. It is unclear whether hydrate polymorphs were counted when calculating hydration probability.
The salt screening experiment, as designed, is not well suited to validate the observed correlation. Unless the model predicts either zero or 100% hydrate probability, the reported correlations between hydrate probability and ESP (Figure 3) cannot be meaningfully supported or rejected regardless of the outcome (from all hydrates to none hydrate).

Minor issues
L65-67, Salts do not always exhibit improved aqueous solubility relative to their non-ionized forms.
L89, spell out API (Application Programming Interface) to avoid confusion with API, referring to active pharmaceutical ingredient elsewhere in the text.

Reviewer #2

(Remarks to the Author)
The manuscript by Holleb et al. provides an interesting, highly practical, and likely impactful contribution related to the solid formulation of pharmaceuticals, agrochemicals, and other organic crystals. The authors' analysis relates the charge delocalization in the ion to the likelihood of hydrate formation, and it is physically rationalized in terms of the electrostatic potential and the strength of water-ion interactions in the crystal. The results are supported by a substantial experimental dataset derived from the Cambridge Structural Database (CSD) and computational analysis of the electrostatic potentials of common counterions. A major strength of the work is the development of a straightforward "rule" for hydrate prediction, which the authors effectively demonstrate through a sample case, making the findings highly accessible to practitioners in the field. The work is important and the paper is written in a very accessible manner, making it a good fit for the broad readership of Nature Comms.

I happily recommend acceptance of this manuscript, with just minor suggestions:

- The aminoacetanilide salt application worked out nicely, but it is worth noting that the probabilities for hydrate formation were low enough in all four salts (13-30%) that there was likely some "luck" involved in the outcome they observed. For example, it wouldn't have been too surprising with those probabilities if they found that none of them formed hydrated salts, or if the HBr salt formed a hydrate when HCl did not, etc. It would be good to explicitly acknowledge that the probabilities computed are useful but imperfect predictors.

- The discussion of water coordination with halide ions was interesting. This section sparked a question regarding the behavior of larger ions: do these more delocalized charges noticeably decrease the propensity for water to coordinate directly with the anionic site? Or is it simply a matter of the water-ion interactions becoming weaker as the charge becomes more delocalized? To address this, the authors might consider looking at the distribution of distances between the water oxygen and the anionic site in their crystal data set.

Reviewer #3

(Remarks to the Author)
I find the results presented by the authors to be both important and interesting. I believe that the Nature Communication be an appropriate medium to present this work to the community. However, there are some issues that need to be addressed or clarified beforehand.
The main question is why authors limited their research to Active Pharmaceutical Ingredients. I understand that the driving force was prediction of hydrate formation in pharma industry, but the problem is much wider since water is the Nemesis of crystal engineering. I believe that if a generalization over all crystals were possible, the impact of the discovery would be much greater. However, if such generalization cannot be made and the findings are only aplicable to counterions used in pharma I suggest that the authors be more transparent about this limitation in the abstract and conclusions.
Moreover, the selection of couterions to investigate the correlation was made based on paper from 2008, which seems to be using Orang Book from 2006. I my opinion in this crutial step Authors should use up-to-date information and present current state of subject i.e #52 (secondary referencing to the same paper) and #121.
Furthermore, given that the selection was based on data nearly two decades old, I would appreciate an explanation as to why only three groups of anions were chosen (skiping for example phospahtes and nitrates) particularly since two of these groups have subsequently merged. The same question can be applied to cations since zinc is skipped.
To a lesser degree, this issue of outdated data is also present in the CSD database used for the survey, which is nearly two years old. It is unclear why a more more recent version was not utilized, especially since it would expand the range of couterions types analyzed (more of them could hit the 100 unique entries treshold).
The data-mining section would also benefit from additional explanations:
• What autors mean by unique entry?
• Why do the numbers presented in the Results section differ from those in the Extended Data Figure S1
While applying the framework in a practical setting is beneficial, the current screening experiment—limited to one cation and four anions—is insufficiently sized. Furthermore, it is unclear why halides were accompanied by γ-resorcylate. The rationale for choosing this specific anion over others requires further clarification.
In my opinion, a comprehensive survey of the database would provide the strongest confirmation of these claims than a limited screening experiment. The cursory data-mining for salts of DABCO is in line with the authors' findings, despite some outliers. To be clear, I do not consider these outliers to be arguments against the paper, as the study discusses a general tendency rather than a rigid rule with 100% accuracy
The manuscript is well-written in a clear and informative fashion; however, I have identified several issues that should be addressed:
• Table 1 and Table 2: for molecules with two pKa values: Which stage of (de)protonation was used for ESP calculation? Is the number of occurrences (NCSD) includes all forms?
• In my opinion, Tables are missing many important counterions like, tartrate, malate, citrate, phosphate, or nitrate.
• Figure 3b: The scale of this subplot should be adjusted to fit the data points more closely. Alternatively, the layout of the entire figure should be modified to minimize unnecessary empty space.
• Figure 4. The pKa values should not be rounded prematurely, as this leads to mathematical inconsistencies; $5-(-6)\neq 12$
In conclusion, I maintain that this discovery is highly significant, not only for the pharm industry but for the chemisty and crystal engineering in general. For this reason, I would strongly advocate for broadening the scope of the survey or, at the very least, addressing the other mentioned issues prior to publication.

Version 1:

Reviewer comments:

Reviewer #1

(Remarks to the Author)
The authors adequately addressed my concerns. It is a good paper to publish.

Reviewer #2

(Remarks to the Author)
The authors have substantially improved the paper by extending the size of their data set, generalizing beyond pharmaceutical APIs, providing more discussion on the practical use of their approach, and generally improving the nuance and caveats surrounding the discussion of the findings and their impact.

I recommend the paper be accepted in its current form.

Reviewer #3

(Remarks to the Author)
I am satisfied with the authors' responses to my review and am pleased that my comments were helpful. However, I still believe that Figure 4 would benefit from adjusting the x-axis limits to the data range in each specific subplot. In my opinion, using identical axis limits for all subplots makes them unnecessarily crowded and leaves a substantial amount of empty space.
I hope that this, more editorial than scientific, issue will be resolved by the Editor and that the paper will be published soon, as the findings are very important and interesting and are presented in a clear and convincing manner.

Open Access This Peer Review File is licensed under a Creative Commons Attribution 4.0 International License, which permits use, sharing, adaptation, distribution and reproduction in any medium or format, as long as you give appropriate credit to the original author(s) and the source, provide a link to the Creative Commons license, and indicate if changes were made.
In cases where reviewers are anonymous, credit should be given to 'Anonymous Referee' and the source.
The images or other third party material in this Peer Review File are included in the article's Creative Commons license, unless indicated otherwise in a credit line to the material. If material is not included in the article's Creative Commons license and your intended use is not permitted by statutory regulation or exceeds the permitted use, you will need to obtain permission directly from the copyright holder.
To view a copy of this license, visit https://creativecommons.org/licenses/by/4.0/

Reviewer #1 (Remarks to the Author):

General comment

This manuscript tackles an important and timely question concerning the hydration tendency of molecular salts. However, the analysis is largely confined to empirical correlations between hydration probability of counterions and their charge density (as quantified by ESP(max/min)), which limits the mechanistic depth of the conclusions.

Major issues

Because water does not distinguish between the API ion and the counterion, considering counterions alone to predict hydration propensity is not justified. Although the hydration propensity of API ions cannot be quantified in the same manner as that of counterions due to the limited number of available salts, this limitation does not justify dismissing the role of the API ion in hydration. Rather, it reflects a constraint of the methodology employed in the present study.

Mechanistic understanding requires treating the ion pair as an integrated entity rather than considering the counterion in isolation. This may help explain why the trends observed for halide salts differ markedly from those of other salts. The current analysis does not capture such ion-pair–specific effects.

We agree that the behavior of a salt ultimately depends on the interplay between the main component and the counterion. While a comprehensive study of all possible main-component variations is not feasible within the scope of this work, we recognize the importance of discussing how this relationship may influence the trends we report. We have expanded the manuscript to make these connections more explicit and to address the limitations inherent in focusing primarily on counterion-driven patterns.

At the same time, we believe that emphasizing the counterion remains a practical and viable approach. In many real-world screening scenarios, the choice of counterion is the variable under consideration, not the main component. Framing our analysis in this way reflects how salt-selection decisions are often made in practice, while still acknowledging the complementary role of the main component. We also find that counterions, are often more charge-dense than the typically larger and more diffuse main-component ions.

In the revised manuscript, we examine several widely studied salt-formers in the CSD to assess whether the broader trends we report are reflected in specific, well-studied examples. This allows us to explicitly discuss the role of interactions between the main component and the counterion. As shown in Figure 5, we include four representative cationic salt formers (including the salts of 4-aminoacetanillinide) and four anionic salt formers to illustrate how the general CSD-wide observations manifest in individual cases with a common main component.

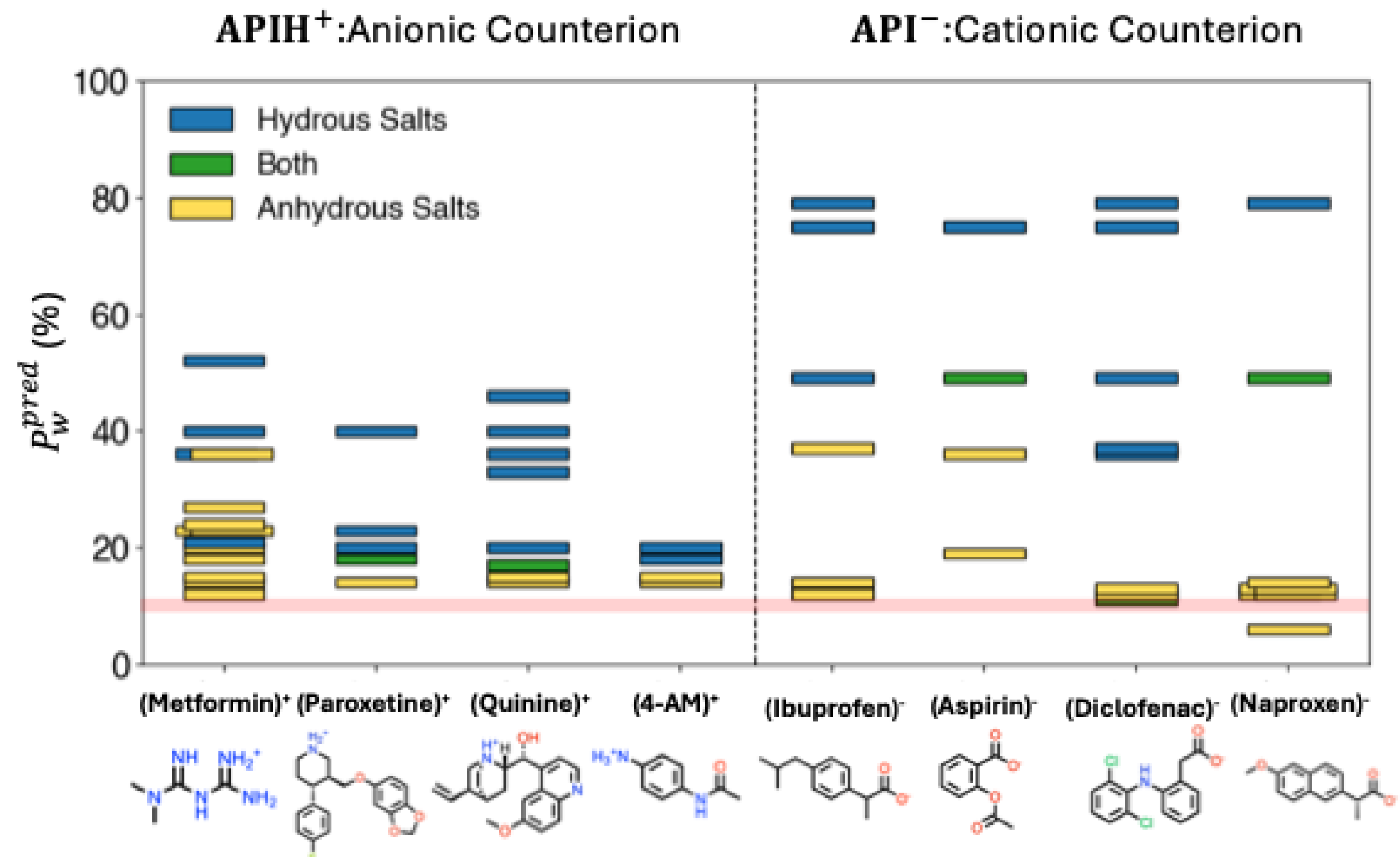

Figure 6 | Occurrence of hydrous and anhydrous salts for four basic (left) and four acidic (right) AMIs with multiple reported salts in the CSD. Each bar represents a crystal structure of an AMI paired with a common counterion. For example, quinine salts span from maleate$^-$ (lowest yellow bar) to sulfate^{2-} (highest blue bar). Hydrated salts are shown in blue, anhydrous salts in yellow, and counterions exhibiting both hydrated and anhydrous forms in green. 4-AM$^+$ refers to salts of 4-aminoacetanilide prepared in the screening experiments. The red line indicates the average P_w^{pred} of the AMIs based on their ESP-derived values and corresponding hydration equations (ammoniums and carboxylates).

To build on our example of 4-AM and further understand the impact of the AMI structure on salt hydration (beyond that of the smaller common counterion from which P_w^{pred} is calculated), we examined hydration trends in salts of eight AMIs (Figure 6). Alongside 4-AM, we selected three basic and four acidic AMIs that form salts with a range of common counterions. We then evaluated whether the predicted salt hydration propensities P_w^{pred} (calculated from the structure of the common counterion alone), translate into consistent behaviour within individual AMI systems.

Across the analyzed examples (Figure 6), there seems to be a hydration threshold in P_w^{pred}, denoted $P_w^{pred,*}[AMI]$, above which hydration of salts generally occurs. Below this limit, predominantly anhydrous salts appear while those above it tend to be hydrous. While the outcome will also depend on crystallization conditions, this observation is useful, as the

formation of an anhydrous salt suggests that salts of counterions with lower values of P_w^{pred} are also likely to be anhydrous.

Although exceptions exist and are expected (e.g. the cinnamate salt of metformin), the identified salts follow the ranking of common counterions. While specific interactions between the AMI and the counterion undoubtedly influence hydration outcomes, the relative ordering of salts based on counterion-derived P_w^{pred} remains congruent with the broader CSD trends. These rankings are effective, as rationalized earlier, because common counterions are typically small and possess localized charges, resulting in more extreme ESP values than those of the larger AMIs typically involved in salt formation. For example, the average P_w^{pred} computed using the ESP extrema of the eight AMIs studied in Figure 6 is approximately 15% (shown as a red band), which is lower than that calculated from the structure of most common counterions reported in Tables 1 and 2. It's also worth noting that many of the AMIs in Figure 6, are relatively simple and small compared to many AMIs. This means that 15% may be an overestimate of the average AMI hydration propensity.

The manuscript also directly acknowledges the limitation in our approach. While the overall trends we report are a useful tool, the precise outcomes remain system-dependent. Despite this limitation, we believe the trends identified from the CSD and exemplified in the test compounds would be of real value to the crystal engineering community and beyond.

Although counterion selection offers significant control over hydration propensity, the threshold $P_w^{pred,*}[AMI]$ remains system-dependent and relies on the interaction between the AMI and the counterion. Exceptions are therefore expected. While complete control over crystallization remains an aspiration, modelling charge density in counterions provides a framework for predicting hydration behavior in molecular salts. Future work exploring the interplay between AMI structure and counterion properties may reveal further insights into the hydration of molecular salts.

CSD may not provide comprehensive data for attaining accurate hydrate propensity for the following reasons:

1. The stability of hydrates depends on a) temperature and b) water activity. Since majority of crystallization experiments were conducted at or near room temperature and many crystallization experiments were performed below water activity of 1, many hydrates are likely missed from the CSD.

The CSD only contains structural data. We do not know the temperature or the activities of any of the crystallizations of the resulting structures in the CSD. This is indeed a major limitation. However, when analyzing large amounts of data (like in our study), things average out. So yes, hydrates are likely missed in the CSD, but they will be equally missed in Chloride salts than benzoate salts so on average all classes of compounds will suffer a “hydrate” loss due to variability in crystallization conditions. Equally, some non-hydrated salts may not be reported so the ‘issue’ also exists for the anhydrous salts.

Although the CSD is inherently incomplete, it remains the most extensive source of data on the outcomes of real crystallization experiments. The Cambridge Structural Database has amassed over a million structures spanning decades, making it not only a highly valuable resource but arguably the only practical avenue for assembling the volume of data required to address this important question. A very comprehensive screening, even discounting the attrition rate of crystallization successes, could not replicate the breadth and quantity of crystallization outcomes the CSD affords access to.

2. Many observed hydrates do not have crystal structures solved and, hence, unavailable in CSD for analysis.

As explained above, this will impact all hydrate types to an equal extent. This will also impact anhydrous salts equally and across different salt types equally.

Because our analysis focuses on the ratio of hydrous structures to the total number of hydrous + anhydrous structures (our probability measure), the overall trends should remain robust even if some hydrous or anhydrous forms have not yet been reported, provided that the dataset is sufficiently large. For this reason, we set a threshold of at least 100 structures before calculating probabilities. As the CSD continues to grow, additional hydrates and anhydrates will undoubtedly be reported, increasing the absolute counts. However, the underlying probabilities are expected to remain stable.

Practically, an infinite number of crystallization outcomes could exist, and many potential hydrous and anhydrous structures will never be solved experimentally or deposited. As the CSD grows, additional structures will certainly appear. However, the existence of an unbounded number of hypothetical, unobserved forms does not diminish the value of the >30k deposited structures in our dataset. These entries represent real crystallization experiments with real outcomes.

To demonstrate the stability of P_w over time, we repeated the same CSD search across multiple database versions. In our current dataset (CSD version 5.45), chloride has 7707 structures and P_w = 31%. Performing the equivalent search in the November 2016 release (version 5.38; N_{CSD} = 4975)

also shows P_w = 31%. The same analysis was then performed using the November 2011 release (version 5.33; N_{CSD} = 3502) showing P_w = 30%. Although the number of chloride salts has more than doubled since 2011, the corresponding probability has remained essentially unchanged for ~15 years. This consistency supports the expectation that, while absolute counts will always continue to increase, P_w remains stable once the sample size is sufficiently large to be representative.

3. It is unclear whether hydrate polymorphs were counted when calculating hydration probability.

We have revised our methods section to clarify that polymorphic salts, and salt hydrates, were included.

> By searching within the best R-factor subset we remove redeterminations while retaining polymorphs of salts and salt hydrates.

The salt screening experiment, as designed, is not well suited to validate the observed correlation. Unless the model predicts either zero or 100% hydrate probability, the reported correlations between hydrate probability and ESP (Figure 3) cannot be meaningfully supported or rejected regardless of the outcome (from all hydrates to none hydrate).

We have rephrased relevant sections to clarify that the model compound was chosen to demonstrate the use of the proposed framework, not as a statistical validation of it. We have also included the section comparing hydration outcomes of different compounds in the CSD to bolster our model system, demonstrating that it does not behave uniquely compared to other similar molecules in the CSD (Figure 6).

Minor issues

L65-67, Salts do not always exhibit improved aqueous solubility relative to their non-ionized forms.

We thank the reviewer for noticing this inaccuracy, we have adjusted the phrasing of the line.

> Notably, around 50% of drugs are salts as this often improves solubility and related properties.

L89, spell out API (Application Programming Interface) to avoid confusion with API, referring to active pharmaceutical ingredient elsewhere in the text.

We thank the reviewer for the suggestion, and we have spelled out API in this context to avoid

confusing the two abbreviations.

> Recent studies making use of the CSD Python Application Programming Interface (CSD Python API), have enabled more complex analyses of hydrates in the CSD, with Werner and Swift reporting structural relationships between hydrate–anhydrate pairs.

Reviewer #2 (Remarks to the Author):

The manuscript by Holleb et al. provides an interesting, highly practical, and likely impactful contribution related to the solid formulation of pharmaceuticals, agrochemicals, and other organic crystals. The authors' analysis relates the charge delocalization in the ion to the likelihood of hydrate formation, and it is physically rationalized in terms of the electrostatic potential and the strength of water-ion interactions in the crystal. The results are supported by a substantial experimental dataset derived from the Cambridge Structural Database (CSD) and computational analysis of the electrostatic potentials of common counterions. A major strength of the work is the development of a straightforward "rule" for hydrate prediction, which the authors effectively demonstrate through a sample case, making the findings highly accessible to practitioners in the field. The work is important and the paper is written in a very accessible manner, making it a good fit for the broad readership of Nature Comms.

I happily recommend acceptance of this manuscript, with just minor suggestions:

- The aminoacetanilide salt application worked out nicely, but it is worth noting that the probabilities for hydrate formation were low enough in all four salts (13-30%) that there was likely some "luck" involved in the outcome they observed. For example, it wouldn't have been too surprising with those probabilities if they found that none of them formed hydrated salts, or if the HBr salt formed a hydrate when HCl did not, etc. It would be good to explicitly acknowledge that the probabilities computed are useful but imperfect predictors.

We certainly agree that some luck was involved in finding a system that behaved ideally for our model and have made note of the fact that our method is imperfect and outliers will be expected.

> While these four salts represent only a portion of every possible salt (and conceivably could have been all anhydrous), it demonstrates how the framework can be applied. If the hydrated bromide salt was obtained first, it would suggest going lower in propensity (as done with iodide and γ-resorcylate) to identify a potential anhydrous salt form.

Although counterion selection offers significant control over hydration propensity, the threshold $P_w^{pred,*}[AMI]$ remains system-dependent and relies on the interaction between the AMI and the counterion. Exceptions are therefore expected. While complete control over crystallization remains an aspiration, modelling charge density in counterions provides a framework for predicting hydration behavior in molecular salts.

In addition to discussing the limitation more directly, we have added a survey of the CSD to contextualize our findings of multiple salts among other commonly studied systems that form multiple salts in Figure 6.

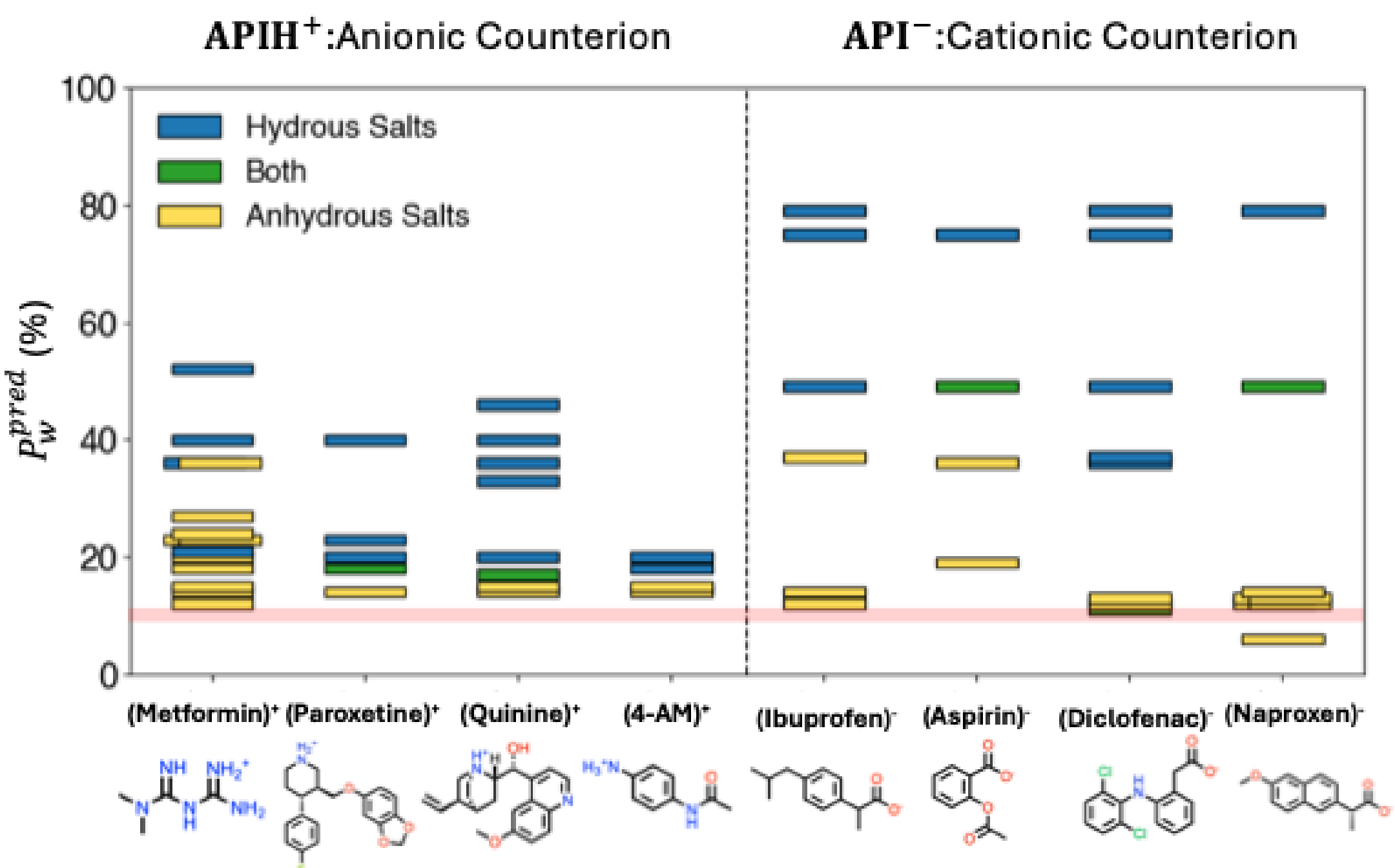

Figure 6 | Occurrence of hydrous and anhydrous salts for four basic (left) and four acidic (right) AMIs with multiple reported salts in the CSD. Each bar represents a crystal structure of an AMI paired with a common counterion. For example, quinine salts span from maleate$^-$ (lowest yellow bar) to sulfate^{2-} (highest blue bar). Hydrated salts are shown in blue, anhydrous salts in yellow, and counterions exhibiting both hydrated and anhydrous forms in green. 4-AM$^+$ refers to salts of 4-aminoacetanilide prepared in the screening experiments. The red line indicates the average P_w^{pred} of the AMIs based on their ESP-derived values and corresponding hydration equations (ammoniums and carboxylates).

To build on our example of 4-AM and further understand the impact of the AMI structure on salt hydration (beyond that of the smaller common counterion from which P_w^{pred} is

calculated), we examined hydration trends in salts of eight AMIs (Figure 6). Alongside 4-AM, we selected three basic and four acidic AMIs that form salts with a range of common counterions. We then evaluated whether the predicted salt hydration propensities P_w^{pred} (calculated from the structure of the common counterion alone), translate into consistent behaviour within individual AMI systems.

Across the analyzed examples (Figure 6), there seems to be a hydration threshold in P_w^{pred}, denoted $P_w^{pred,*}[AMI]$, above which hydration of salts generally occurs. Below this limit, predominantly anhydrous salts appear while those above it tend to be hydrous. While the outcome will also depend on crystallization conditions, this observation is useful, as the formation of an anhydrous salt suggests that salts of counterions with lower values of P_w^{pred} are also likely to be anhydrous.

Although exceptions exist and are expected (e.g. the cinnamate salt of metformin), the identified salts follow the ranking of common counterions. While specific interactions between the AMI and the counterion undoubtedly influence hydration outcomes, the relative ordering of salts based on counterion-derived P_w^{pred} remains congruent with the broader CSD trends. These rankings are effective, as rationalized earlier, because common counterions are typically small and possess localized charges, resulting in more extreme ESP values than those of the larger AMIs typically involved in salt formation. For example, the average P_w^{pred} computed using the ESP extrema of the eight AMIs studied in Figure 6 is approximately 15% (shown as a red band), which is lower than that calculated from the structure of most common counterions reported in Tables 1 and 2. It's also worth noting that many of the AMIs in Figure 6, are relatively simple and small compared to many AMIs. This means that 15% may be an overestimate of the average AMI hydration propensity.

- The discussion of water coordination with halide ions was interesting. This section sparked a question regarding the behavior of larger ions: do these more delocalized charges noticeably decrease the propensity for water to coordinate directly with the anionic site? Or is it simply a matter of the water-ion interactions becoming weaker as the charge becomes more delocalized? To address this, the authors might consider looking at the distribution of distances between the water oxygen and the anionic site in their crystal data set.

Thank you for the suggestion. We have investigated the water-O^- distances in several of our datasets. Generally, we find the hydrogen bond distances cluster around similar bond lengths. While we expect that the electrostatics will still affect interaction strength, the hydrogen bonding parameters are quite similar. We have included these distributions in our ESI as figure S8.

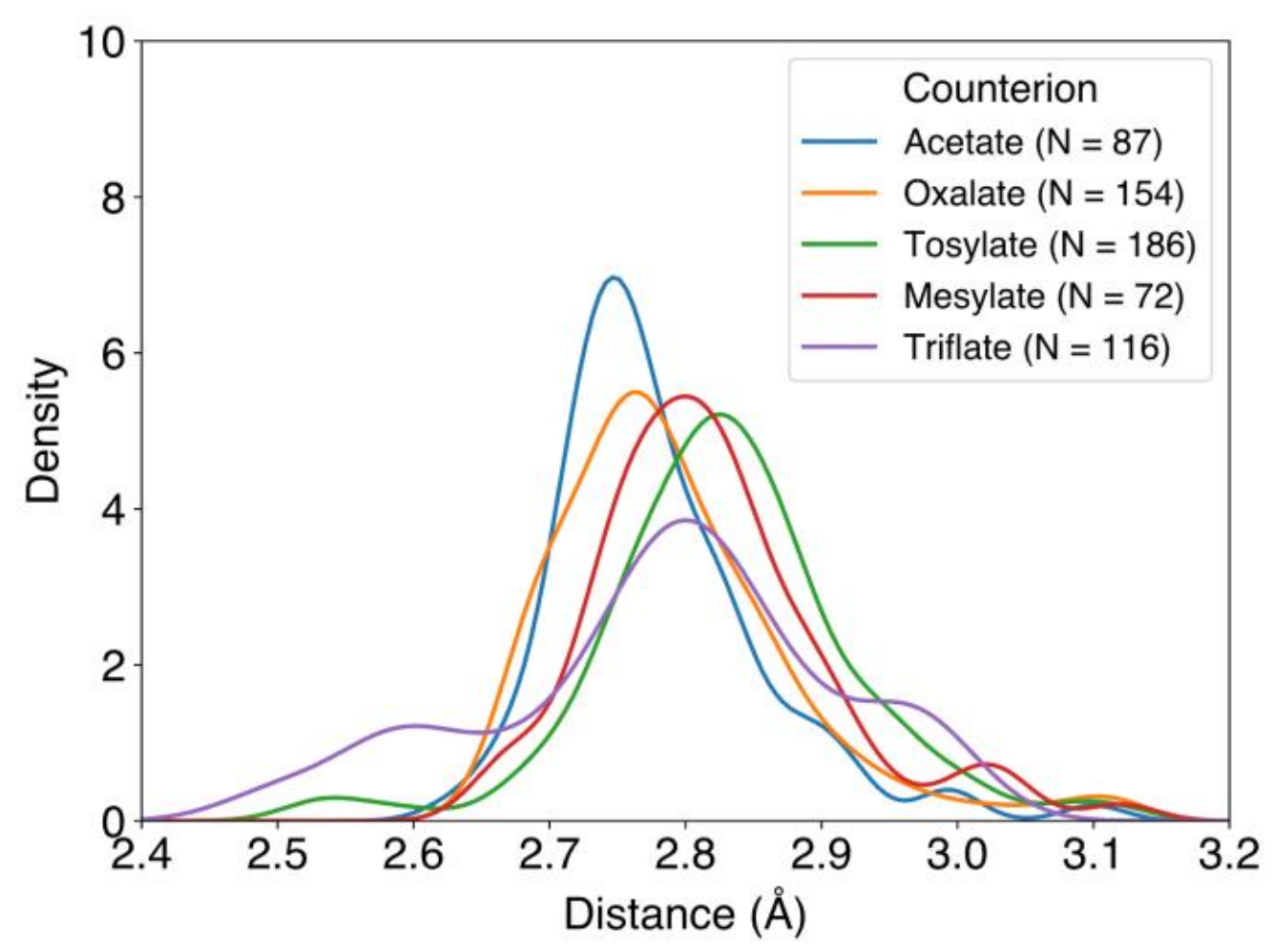

Figure S8. Distribution of water-oxyanion distances in the acetate, mesylate, tosylate, oxalate, and triflate datasets. The number represents the number of interactions included (a structure with three water-O^- contacts will contribute 3).

Reviewer #3 (Remarks to the Author):

I find the results presented by the authors to be both important and interesting. I believe that the Nature Communication be an appropriate medium to present this work to the community. However, there are some issues that need to be addressed or clarified beforehand.
The main question is why authors limited their research to Active Pharmaceutical Ingredients. I understand that the driving force was prediction of hydrate formation in pharma industry, but the problem is much wider since water is the Nemesis of crystal engineering. I believe that if a generalization over all crystals were possible, the impact of the discovery would be much greater. However, if such generalization cannot be made and the findings are only aplicable to counterions used in pharma I suggest that the authors be more transparent about this limitation in the abstract and conclusions.

We thank the reviewer for the comment. While we chose to highlight the relevance of our findings to pharmaceutical formulation, we do not believe our findings are confined to this context. To broaden our scope, we conducted a survey of the most common counterions in the CSD to gather all those with >100 CSD entries regardless of their use. We then regrouped these counterions by family and analyze the hydration trends across the CSD.

Through this comprehensive screening, we have roughly doubled the number of counterions included (from 26 to 49) and increased the number of CSD entries analyzed by ~1/3 (from 21k to 31k). Every counterion we could identify that met the criteria (no intermolecular hydrogen bond donors, accessible charged site, >100 CSD entries) were included regardless of its application. We also present all counterions plotted together in the ESI (Figure S3) to highlight that while grouping by family improves the correlation (Figure 4), the trend holds across a wide, structurally diverse set of 49 cations and anions in the CSD.

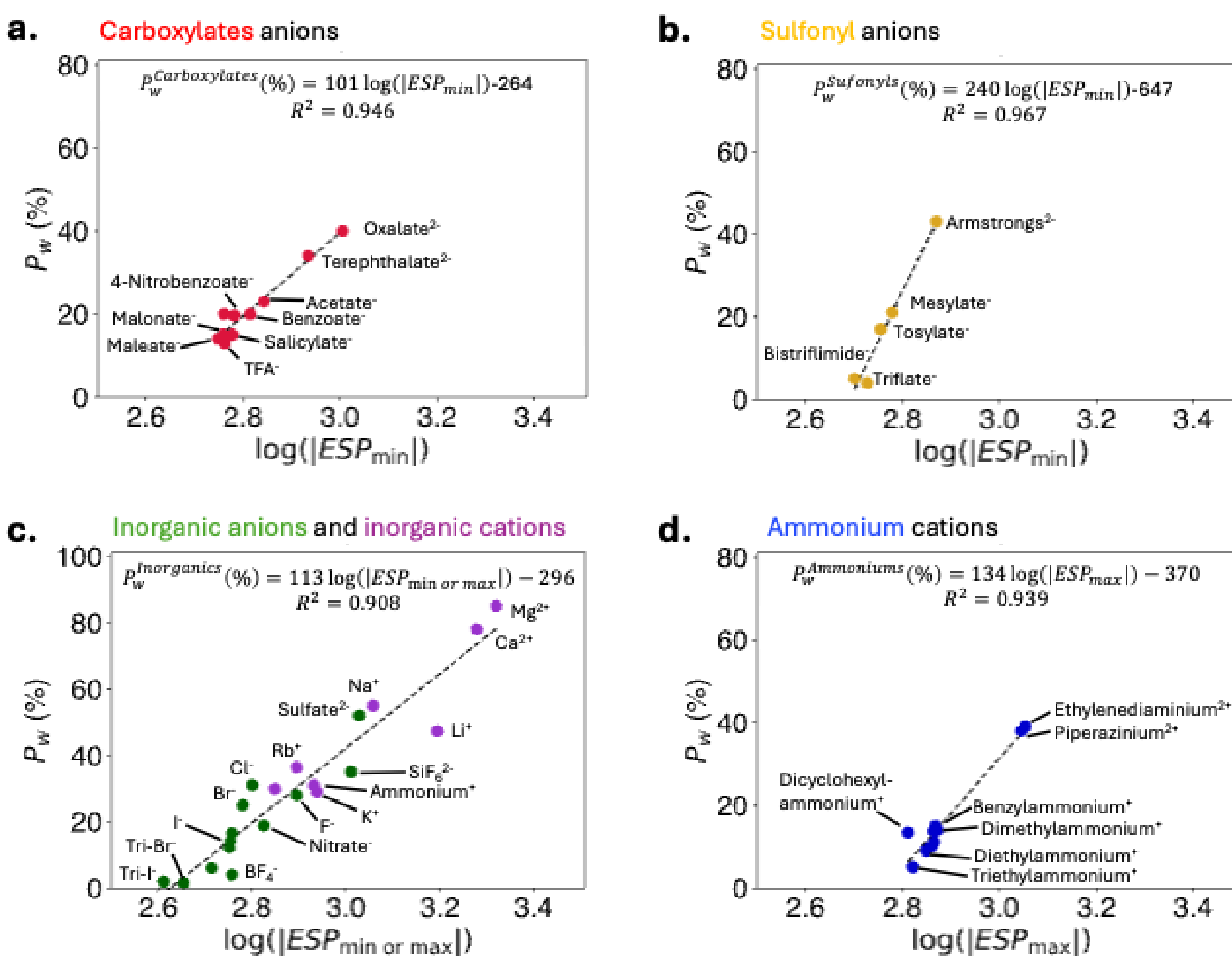

Figure 4 | Correlation between electrostatic potential (ESP) extrema and hydration propensity. Relationship between ESP extrema and P_w for **salts containing common counterions grouped by family: (a) carboxylate anions, (b) sulfonyl anions, (c) inorganic ions, and (d) ammonium cations. Selected counterions are labeled for illustration; full datasets are provided in Table 1 of the ESI.**

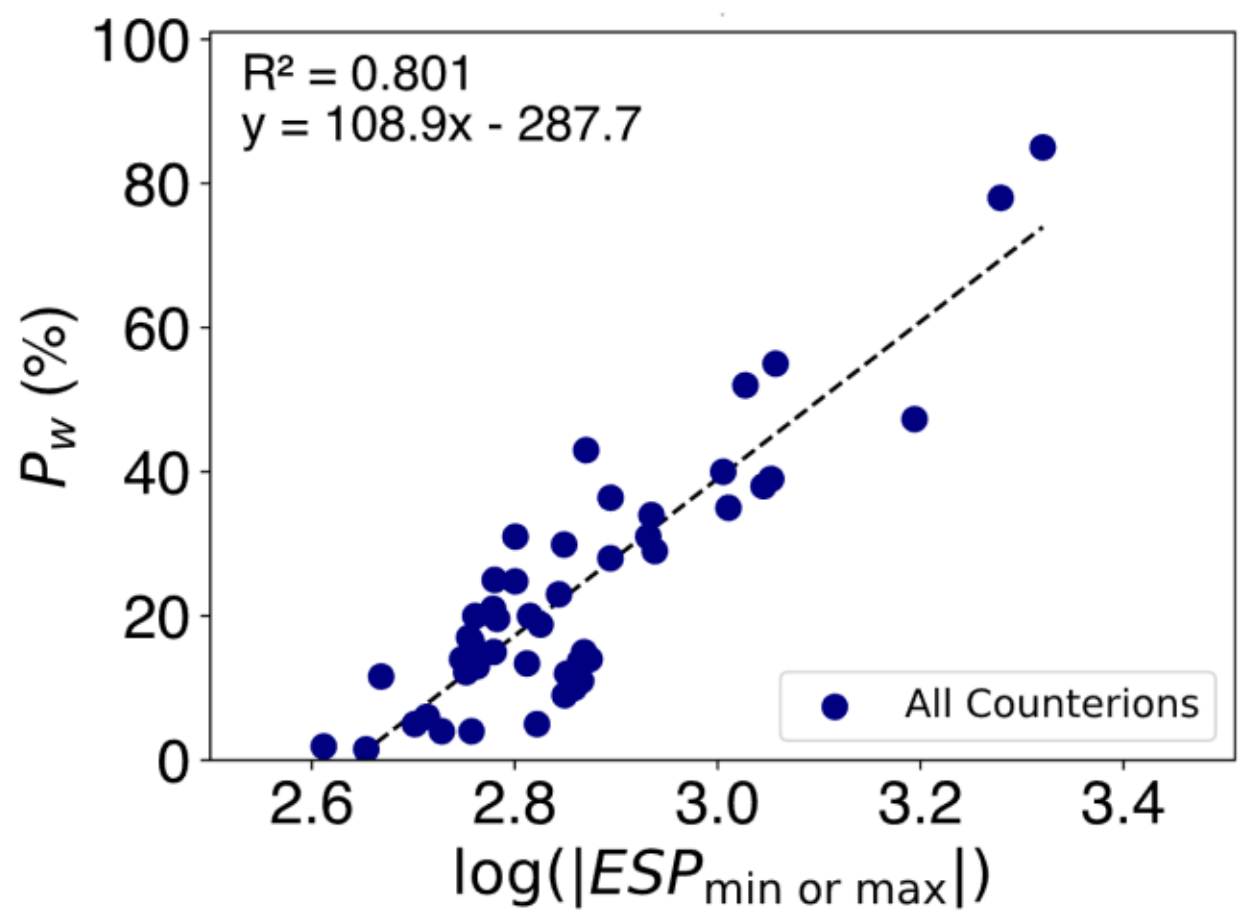

Extended Data Figure S3| Scatterplot of ESP minimum or maximum vs. P_w for all 49 counterions.

Moreover, the selection of couterions to investigate the correlation was made based on paper from 2008, which seems to be using Orang Book from 2006. I my opinion in this crutial step Authors should use up-to-date information and present current state of subject i.e #52 (secondary referencing to the same paper) and #121.
Furthermore, given that the selection was based on data nearly two decades old, I would appreciate an explanation as to why only three groups of anions were chosen (skiping for example phospahtes and nitrates) particularly since two of these groups have subsequently merged. The same question can be applied to cations since zinc is skipped.

Through our new screening, we capture all counterions with enough structure retrievable by SMILES string regardless of their appearance in the Orange Book. We have also included Nitrate among the inorganics family.

As we had previously found, counterions with extra intermolecular donors tend to behave in ways that are less predictable, and hydrogen phosphate and dihydrogen phosphate are the only ionization states with enough structures and were not included. We also agree that phosphates, tartrate, and citrate are three important ions, however, they each contain intermolecular hydrogen bond donors. We add a more thorough discussion of this limitation in the manuscript and plot the outlier counterions with donors in the ESI.

> Salts in the CSD were surveyed, and common counterions were identified and classified into families. All counterions appearing in at least 100 crystal structures were extracted by clustering based on SMILES representations, resulting in 71 common counterions split into 4 types (Figure S1). Of these, 22 were excluded from further analysis because they were either (i) too bulky to allow direct interaction between water and the charged site (6 ions, e.g. tetrabutylammonium), or (ii) contained additional (non-intra molecular as in maleate)

hydrogen-bond donors beyond the charged XH^+ group (16 ions, e.g. citrate), leading to more complex and less predictable hydration behaviour. The remaining 49 counterions were considered for analysis, of which 46 were classified into five distinct families, with three left unclassified (pyridinium, picrate, and DMAP). This yielded a dataset of 46 common counterions across more than 31,000 molecular salts, grouped as inorganic anions (13 counterions, with thiocyanate considered as an inorganic due to its similarity to iodide), sulfonyl anions (5), carboxylate anions (10), inorganic cations (8), and ammonium cations (10), with all counterions analysed shown in Figure 2.

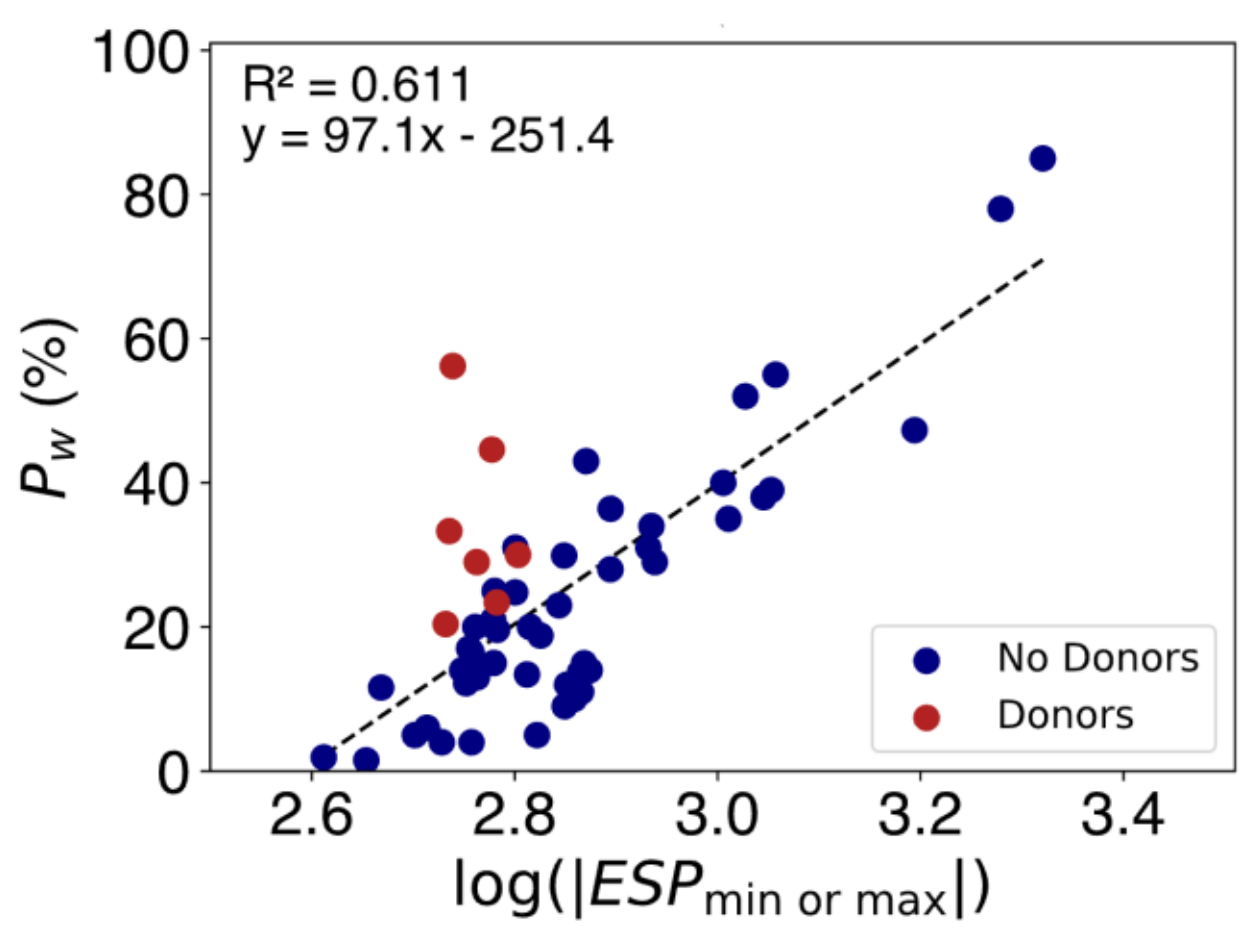

Figure S2| Experimental P_w versus log|$ESP_{min\ or\ max}$| for counterions including anions with intermolecular donors with N_{CSD} > 100 plotted in red.

We have added all alkali and alkaline earth metals that have enough structures. Zinc, however, lies outside the organic subset of the CSD and was therefore not included in the analysis.

To a lesser degree, this issue of outdated data is also present in the CSD database used for the survey, which is nearly two years old. It is unclear why a more more recent version was not utilized, especially since it would expand the range of couterions types analyzed (more of them could hit the 100 unique entries treshold).

The analysis was conducted earlier; however, the manuscript was drafted over the course of a year. However, we believe the statistics we found should still be mostly up to date and that P_w is a durable descriptor. We performed an example search of chloride from 2024, 2016, and 2011 demonstrate that although the precise numbers will change over time, we expect the trends should remain the same.

Chloride:

> version 5.45 (N_{CSD} = 7707, P_w = 31%), November 2016 version 5.38 (N_{CSD} = 4975, P_w = 31%), November 2011 version 5.33 (N_{CSD} = 3502, P_w = 30%).

Despite the number of chloride structures more than doubling since 2011, the calculate P_w has remained stable over ~15 year.

The data-mining section would also benefit from additional explanations:
• What autors mean by unique entry?

When we discuss unique entries, we refer to a dataset with redeterminations removed. We have clarified this in the manuscript

> An initial element filter reduced the 746,345 unique entries (excluding redeterminations while retaining polymorphs) in the *best R-factor* subset to **395,494 organic entries**.

• Why do the numbers presented in the Results section differ from those in the Extended Data Figure S1.

We thank the reviewer for catching our error. Values from a previous dataset were used and not updated. The numbers should now be consistent across the manuscript.

While applying the framework in a practical setting is beneficial, the current screening experiment—limited to one cation and four anions—is insufficiently sized.
Furthermore, it is unclear why halides were accompanied by γ-resorcylate. The rationale for choosing this specific anion over others requires further clarification.
In my opinion, a comprehensive survey of the database would provide the strongest confirmation of these claims than a limited screening experiment. The cursory data-mining for salts of DABCO is in line with the authors' findings, despite some outliers. To be clear, I do not consider these outliers to be arguments against the paper, as the study discusses a general tendency rather than a rigid rule with 100% accuracy

We agree that while the experimental data presents an example use of the framework, it is insufficient to statistically validate the model. We have also included an analysis of salts of commonly studied molecules in the CSD (Figure 6). We find that the trends we observe in the CSD generally hold within specific cases, but as would be expected, there are outliers.

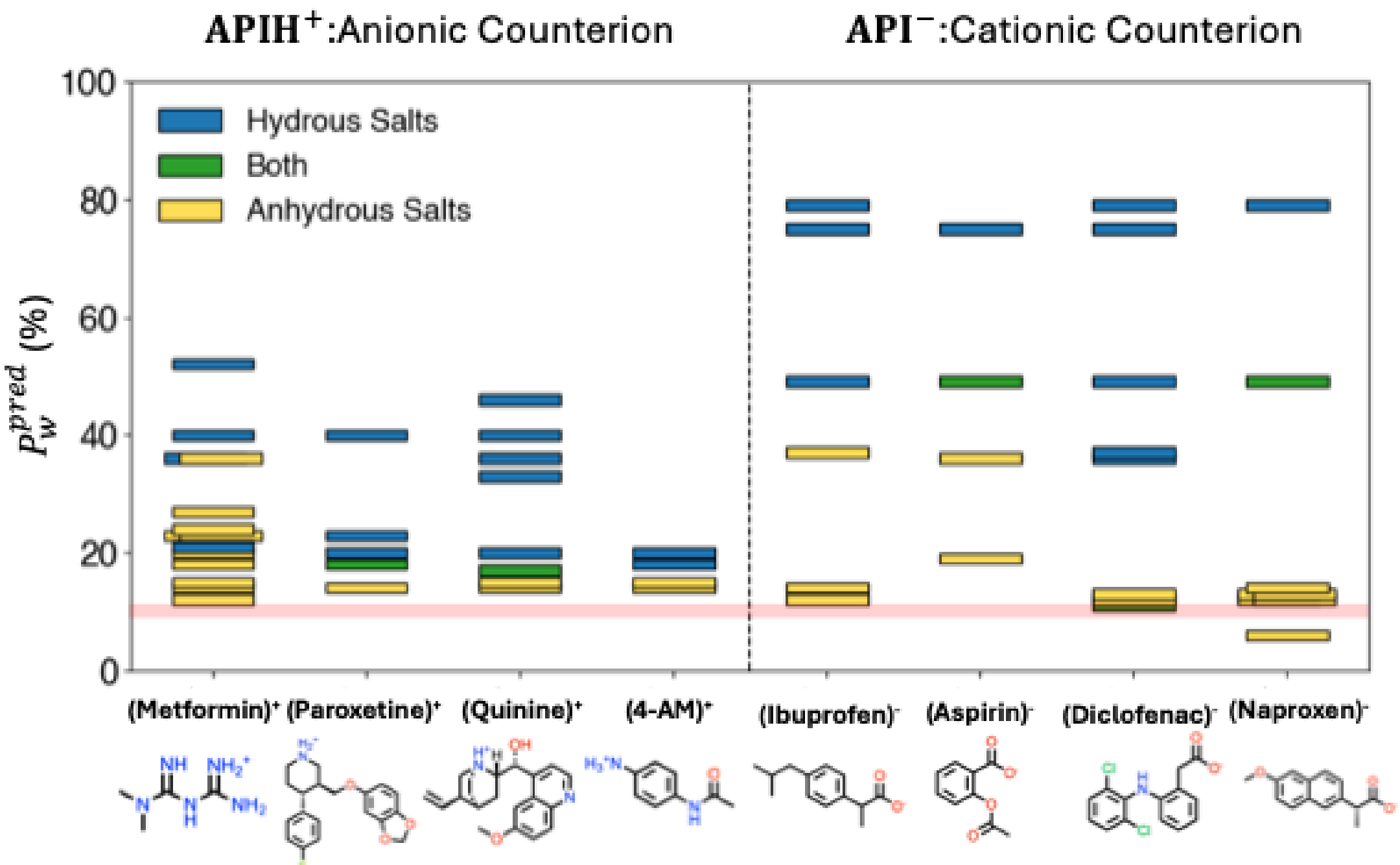

Figure 6 | Occurrence of hydrous and anhydrous salts for four basic (left) and four acidic (right) AMIs with multiple reported salts in the CSD. Each bar represents a crystal structure of an AMI paired with a common counterion. For example, quinine salts span from maleate⁻ (lowest yellow bar) to sulfate²⁻ (highest blue bar). Hydrated salts are shown in blue, anhydrous salts in yellow, and counterions exhibiting both hydrated and anhydrous forms in green. 4-AM⁺ refers to salts of 4-aminoacetanilide prepared in the screening experiments. The red line indicates the average P_w^{pred} of the AMIs based on their ESP-derived values and corresponding hydration equations (ammoniums and carboxylates).

To build on our example of 4-AM and further understand the impact of the AMI structure on salt hydration (beyond that of the smaller common counterion from which P_w^{pred} is calculated), we examined hydration trends in salts of eight AMIs (Figure 6). Alongside 4-AM, we selected three basic and four acidic AMIs that form salts with a range of common counterions. We then evaluated whether the predicted salt hydration propensities P_w^{pred} (calculated from the structure of the common counterion alone), translate into consistent behaviour within individual AMI systems.

Across the analyzed examples (Figure 6), there seems to be a hydration threshold in P_w^{pred}, denoted $P_w^{pred,*}[AMI]$, above which hydration of salts generally occurs. Below this limit, predominantly anhydrous salts appear while those above it tend to be hydrous. While the outcome will also depend on crystallization conditions, this observation is useful, as the

formation of an anhydrous salt suggests that salts of counterions with lower values of P_w^{pred} are also likely to be anhydrous.

Although exceptions exist and are expected (e.g. the cinnamate salt of metformin), the identified salts follow the ranking of common counterions. While specific interactions between the AMI and the counterion undoubtedly influence hydration outcomes, the relative ordering of salts based on counterion-derived P_w^{pred} remains congruent with the broader CSD trends. These rankings are effective, as rationalized earlier, because common counterions are typically small and possess localized charges, resulting in more extreme ESP values than those of the larger AMIs typically involved in salt formation. For example, the average P_w^{pred} computed using the ESP extrema of the eight AMIs studied in Figure 6 is approximately 15% (shown as a red band), which is lower than that calculated from the structure of most common counterions reported in Tables 1 and 2. It's also worth noting that many of the AMIs in Figure 6, are relatively simple and small compared to many AMIs. This means that 15% may be an overestimate of the average AMI hydration propensity.

We have also added more explanation to justify the 4 counterions that were paired with 4-aminoacetanillinide. All salts that were successfully crystallized of 4-AM are reported.

4-AM's weak basicity limited the number of viable acids as many carboxylic acids are not strong enough to protonate the primary amine.

The manuscript is well-written in a clear and informative fashion; however, I have identified several issues that should be addressed:

• Table 1 and Table 2: for molecules with two pKa values: Which stage of (de)protonation was used for ESP calculation? Is the number of occurrences (NCSD) includes all forms?

• In my opinion, Tables are missing many important counterions like, tartrate, malate, citrate, phosphate, or nitrate.

We thank the reviewer for the comment. All the ESP calculations are based on the charge specified in the table. We have added a line to the table headers to make this less ambiguous:

Table 1. Collection of acids and their acid strengths (pK_a), together with the corresponding anions, their family classification (fam), number of CSD observations (N_{CSD}), computed ESP_{min} values, and predicted salt hydration propensities (P_w^{pred}). The equation used to calculate P_w^{pred} depends on the anion family classification, where C = carboxylates, S = sulfonyl anions, I = inorganic anions, and CSD means the P_w was obtained directly from the CSD. The table is ranked by anions from highest to lowest predicted P_w^{pred}. Where multiple acidic groups are present, multiple pK_a values are reported, with the corresponding ESP and hydration propensity calculated for the specific anion shown.

Table 2. Collection of bases and the acid strengths of their conjugated protonated base (pK_a), together with the corresponding cations, their family classification (fam), number of CSD observations (N_{CSD}), computed ESP_{max} values, and predicted salt hydration propensities (P_w^{pred}). The equation used to calculate

P_w^{pred} depends on the cation family classification, where I = inorganic anions, A = ammonium cations, and CSD means the P_w was obtained directly from the CSD. The table is ranked by anions from highest to lowest predicted P_w^{pred}. Where multiple basic groups are present, multiple pK_a values are reported for the conjugated bases, with the corresponding ESP and hydration propensity calculated for the specific cation shown.

• Figure 3b: The scale of this subplot should be adjusted to fit the data points more closely. Alternatively, the layout of the entire figure should be modified to minimize unnecessary empty space.

The Figure containing the regressions has been updated. We have cropped the y axis of the ammoniums, carboxylates, and sulfonyl anions to frame the data more clearly.

• Figure 4. The pKa values should not be rounded prematurely, as this leads to mathematical inconsistencies; 5-(-6)≠ 12

We thank the reviewer for catching our error, this has been corrected in the manuscript.

In conclusion, I maintain that this discovery is highly significant, not only for the pharm industry but for the chemisty and crystal engineering in general. For this reason, I would strongly advocate for broadening the scope of the survey or, at the very least, addressing the other mentioned issues prior to publication.

We thank the reviewer for the comment. We hope our comprehensive analysis of the hydration trends among the most common counterions will help make our findings broad and valuable to the *Nature Communications* readership.
